# Mitochondrial Ultrastructure Is Coupled to Synaptic Performance at Axonal Release Sites

**DOI:** 10.1523/ENEURO.0390-17.2018

**Published:** 2018-01-29

**Authors:** Csaba Cserép, Balázs Pósfai, Anett Dóra Schwarcz, Ádám Dénes

**Affiliations:** Momentum Laboratory of Neuroimmunology, Institute of Experimental Medicine, Hungarian Academy of Sciences, Budapest 1083, Hungary

**Keywords:** crista, cytochrome-c, electron tomography, mitochondria, super-resolution microscopy, synapse

## Abstract

Mitochondrial function in neurons is tightly linked with metabolic and signaling mechanisms that ultimately determine neuronal performance. The subcellular distribution of these organelles is dynamically regulated as they are directed to axonal release sites on demand, but whether mitochondrial internal ultrastructure and molecular properties would reflect the actual performance requirements in a synapse-specific manner, remains to be established. Here, we examined performance-determining ultrastructural features of presynaptic mitochondria in GABAergic and glutamatergic axons of mice and human. Using electron-tomography and super-resolution microscopy we found, that these features were coupled to synaptic strength: mitochondria in boutons with high synaptic activity exhibited an ultrastructure optimized for high rate metabolism and contained higher levels of the respiratory chain protein cytochrome-c (CytC) than mitochondria in boutons with lower activity. The strong, cell type-independent correlation between mitochondrial ultrastructure, molecular fingerprints and synaptic performance suggests that changes in synaptic activity could trigger ultrastructural plasticity of presynaptic mitochondria, likely to adjust their performance to the actual metabolic demand.

## Visual Abstract

**Figure:**
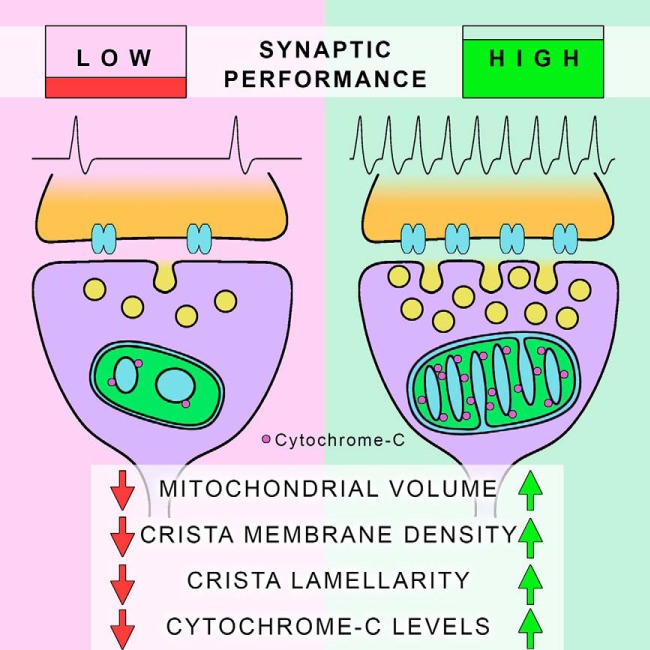

## Significance Statement

Neuronal networks are highly dynamic structures: cellular morphology and synaptic strength is changing as information is processed and stored. This means that the demand for mitochondrial supply is changing in space and time in neurons. Since the architecture ultimately determines the output performance of these organelles, we hypothesized that the ultrastructure of axonal mitochondria could well be a major parameter of adjusting their performance to the actual energetic needs of the synapse. Our results, describing a cell type-independent and synapse-specific correlation between mitochondrial ultrastructure, mitochondrial molecular fingerprints, and synaptic performance, highlight the importance of activity-dependent ultrastructural plasticity of these organelles in neurons and suggest an even more prominent role for mitochondria in neuroplasticity than it was thought previously.

## Introduction

About one-tenth of total gray matter volume is occupied by mitochondria, the powerhouses of cells (Toussaint and Kugler, 1989). These organelles form a rapidly changing, dynamic network in neurons (Barnhart, 2016). As primary energy generators, precise regulation of mitochondrial performance is fundamental for proper functioning of the CNS, due to the exceptionally high ATP consumption of neuronal tissue. Mitochondria are also key regulators of intracellular calcium levels (Gunter et al., 2000; Hall et al., 2012) and contribute to signaling processes, the regulation of cell proliferation, migration, neuronal morphology, and cell viability (Chandel, 2014). Since the main energy consumer in the CNS is synaptic transmission (Harris et al., 2012), intact mitochondrial ATP production, together with calcium regulation, is crucial for proper synaptic function and neuroplasticity (Billups and Forsythe, 2002; Verstreken et al., 2005; Gazit et al., 2016; Smith et al., 2016). Furthermore, the availability of presynaptic mitochondria at axonal release sites directly influences synaptic function (Sun et al., 2013; Rangaraju et al., 2014; Smith et al., 2016).

In highly dynamic neuronal networks cellular morphology and synaptic strength is changing in an activity dependent manner (Muller et al., 2002), resulting in altered neuronal demand for mitochondrial supply in space and time. Recent data also suggest that mitochondrial distribution within neurons is dynamically regulated to match the actual needs. *In vitro* studies confirmed, that motile mitochondria in dendrites as well as in axons are recruited and anchored to synapses whenever the local need for mitochondrial performance increases (Li et al., 2004; Sheng, 2014). It has also been shown in non-neuronal tissues that the inner ultrastructure of mitochondria determines their output performance. The surface area of crista membrane (CM) in a given mitochondrial volume strongly correlates with mitochondrial oxygen consumption and cytochrome oxidase activity (Else et al., 2004; Perkins et al., 2003, 2012; Sood et al., 2014). In addition to CM density, the shape of cristae has also been shown to directly regulate mitochondrial performance output (Cogliati et al., 2013), and influence mitochondrial Ca^2+^ dynamics (Mannella et al., 2013).

Based on these data, we hypothesized that the ultrastructure of these organelles may be shaped to match the actual local needs in neurons. To test this idea with high-resolution imaging, we performed electron tomography and stochastic optical reconstruction microscopy (STORM) super-resolution microscopy studies on mouse brain tissues, combined with serial-section transmission electron microscopy (EM) studies on postmortem human tissue samples. We examined presynaptic mitochondria in GABAergic and glutamatergic axons of the hippocampal formation. We found that CM density, crista lamellarity and cytochrome-c (CytC) density was higher in presynaptic mitochondria of the highly active fast-spiking parvalbumin (PV)-positive basket cells than in the mitochondria of the slow firing type-1 cannabinoid receptor (CB1R)-positive basket cells, which are the well-characterized archetypes of high-activity and low-activity interneurons, respectively. We also examined presynaptic mitochondria in the glutamatergic boutons of the perforant-path terminating zone in the dentate gyrus, and found a strong, cell type-independent correlation between mitochondrial volume, CM density, crista lamellarity, CytC density, and synaptic strength. The results from human postmortem tissue also confirmed that this phenomenon is evolutionally conserved. Our data provide evidence for the logical assumption, that “stronger synapses use stronger mitochondria,” and point out the possibility of the activity-dependent ultrastructural plasticity of these organelles.

## Materials and Methods

### Samples and tissue preparation

All experimental procedures were in accordance with the guidelines set by the Animal Care and Use Committee of the Institute of Experimental Medicine, Hunagarian Academy of Sciences. Six male C57BL/6 (RRID:IMSR_JAX:000664) mice and four CB1R-knockout (CB1R-KO; Zimmer et al., 1999) mice (30–42 d old) were anesthetized with inhalation of isoflurane, followed by intraperitoneal injection of 0.05–0.1 ml of an anesthetic mixture (containing 8.3 mg/ml ketamine, 1.7 mg/ml xylazin hydrochloride, and 0.8 mg/ml promethazinium chloride). Transcardial perfusions were performed in three different ways, depending on the type of experiment that followed. Animals used for immunofluorescent reactions [two wild-type (WT) and two CB1R-KO mice for confocal scanning laser microscopy and STORM super-resolution microscopy] were perfused with 0.9% NaCl solution for 1 min, followed by 4% freshly depolymerized paraformaldehyde (PFA) in 0.1 M phosphate buffer (PB), pH 7.4, for 40 min, and finally with 0.1 M PB for 10 min to wash the fixative out. For the immunogold reactions, we perfused other two WT and two CB1R-KO mice with saline for 1 min, followed by 2% PFA and 1% glutaraldehyde (GA) in 0.1 M Na acetate buffer (pH 6) for 3 min, and then 2% PFA plus 1% GA in 0.1 M borate buffer (pH 8.5) for 40 min. In this case, the fixative was not washed out, but brains were removed, and postfixed overnight in the same fixative at 4°C. For the experiments without immunostaining, we perfused two WT animals with saline for 1 min, followed by 2% PFA and 2% GA in 0.1 M Na acetate buffer (pH 6) for 3 min, and then 2% PFA plus 2% GA in 0.1 M borate buffer (pH 8.5) for 60 min. In this case again, the fixative was not washed out, but brains were removed and postfixed overnight in the same fixative at 4°C. Blocks containing the dorsal hippocampi were dissected and coronal sections were prepared on a vibratome (VT1200S, Leica) at 20-µm thickness for STORM experiments, 50-µm thickness for immunofluorescent experiments, and 60-µm thickness for EM and electron tomography. Control human hippocampal tissue was obtained from one female (59 years old) and one male (55 years old) subjects who died from causes not linked to brain diseases and did not have a history of neurologic disorders. The subjects were processed for autopsy in St. Borbála Hospital, Tatabánya, Department of Pathology. Informed consent was obtained for the use of brain tissue and for access to medical records for research purposes. Tissue was obtained and used in a manner compliant with the Declaration of Helsinki. All procedures were approved by the Regional Institutional Committee of Science and Research Ethics of Scientific Council of Health (ETT TUKEB 31443/2011/EKU [518/PI/11]). Brains were removed 4-5 h after death. The internal carotid and the vertebral arteries were cannulated, and the brain was perfused first with physiologic saline (using a volume of 1.5 l in 30 min) containing heparin (5 ml), followed by a fixative solution containing 4% PFA, 0.05% GA, and 0.2% picric acid (v/v) in 0.1 M PB, pH 7.4 (4–5 l in 1.5–2 h). The hippocampus was removed from the brain after perfusion, and was postfixed overnight in the same fixative solution, except for GA, which was excluded. Blocks were dissected, and 60-µm-thick sections were prepared on a vibratome (VT1200S, Leica).

### Primary antibodies

To detect CB1Rs, we used either a goat polyclonal antibody or a rabbit polyclonal antibody (gifts from Prof. Ken Mackie). For the staining of PV, we used a rabbit polyclonal antibody (PV27, Swant, RRID:AB_2631173). The specificity of this antibody was tested on PV-KO tissue (Swant). To label GABAergic boutons, we used a KO-verified guinea-pig anti-vesicular GABA transporter (vGAT) antibody (131004, Synaptic Systems, RRID:AB_887873). To visualize CytC, we used a mouse monoclonal antibody (clone 6H2.B4, 612302, Biolegend, RRID:AB_315775). The specificity of this antibody has been previously tested and described (Gulyás et al., 2006). To detect TOM20, we used a rabbit polyclonal antibody (Santa Cruz Biotechnology, catalog number s-11415, RRID:AB_2207533; Barna et al., 2016). To label glutamatergic presynaptic boutons, we used a polyclonal guinea-pig antibody, raised against a synthetic peptide from rat vGluT1 (VG1), which peptide does not overlap with the sequence of vGluT2; (Millipore, AB5905, RRID:AB_2301751). The specificity of this antibody has also been described, and it gave identical staining with a well-characterized rabbit anti-VG1 antibody (Melone et al., 2005). A rabbit polyclonal antibody (Synaptic Systems, 160003, RRID:AB_887730), raised against the 1–186 amino acid residues of Homer1 was used to label the postsynaptic density of glutamatergic synapses. This antibody stains selectively the postsynaptic density of glutamatergic synapses (Andreska et al., 2014).

All antibodies gave the previously described and thus expected staining patterns for their respective epitopes. For further control of our stainings, we extensively tested the possible cross-reactivity of the fluorescent secondary antibodies used in multiple labeling experiments. No cross-reactivity was found in any of the cases. Selective labeling, resembling that obtained with the specific antibodies, could not be detected if primary antibodies were omitted.

### Immunofluorescent labeling and confocal laser-scanning microscopy (CLSM)

Before the immunofluorescent staining, the 50-µm-thick sections were washed in PB and Tris-buffered saline (TBS; 0,05 M Tris and 0,9% NaCl; pH 7.4). This was followed by blocking for 1 h in 1% human serum albumin (HSA) and 0.1% Triton X-100 dissolved in TBS. After this, sections were incubated in mixtures of primary antibodies: (1) rabbit and goat anti-CB1R, both diluted in TBS 1:500; or (2) rabbit anti-PV, 1:2000; goat anti-CB1R, 1:500; and guinea-pig anti-vGAT, 1:2000; overnight at room temperature. After incubation, sections were washed in TBS, and sections were incubated overnight at 4°C in the mixture of (1) Alexa Fluor 488-conjugated donkey-anti-rabbit (1:500, Life Technologies) and Alexa Fluor 594-conjugated donkey-anti-goat (1:500, Invitrogen) antibodies; or (2) Alexa Fluor 488-conjugated donkey-anti-rabbit (1:500, Life Technologies), Alexa Fluor 594-conjugated donkey-anti-goat (1:500, Invitrogen) and Alexa Fluor 647-conjugated donkey-anti-guinea-pig (1:500, Jackson ImmunoResearch), all diluted in TBS. Secondary antibody incubation was followed by washes in TBS, PB, and the sections were treated with DAPI for 1.5 h (1:10,000, in PB). Finally, we washed the sections, mounted them onto glass slides, and coverslipped with Aqua-Poly/Mount (Polysciences). Immunofluorescence was analyzed using a Nikon Eclipse Ti-E inverted microscope (Nikon Instruments Europe B.V.), with a CFI Plan Apochromat VC 60XH oil immersion objective (numerical aperture: 1.4) and an A1R laser confocal system. We used 405-, 488-, 561-, and 642-nm lasers (CVI Melles Griot), and scanning was done in line serial mode. Image stacks were obtained with NIS-Elements AR software, and deconvolved using Huygens Professional software (www.svi.nl).

### STORM super-resolution microscopy

Before the immunofluorescent staining for the super-resolution experiments, the 20-µm-thick sections were washed in PB and TBS. This was followed by blocking for 1 h in 1% HSA dissolved in TBS. After this, sections were incubated in mixtures of primary antibodies (1) goat anti-CB1R, 1:500; rabbit anti-PV, 1:2000; and mouse anti-CytC, 1:3000; (2) rabbit anti-TOM20, 1:500 and mouse anti-CytC, 1:3000; or (3) guinea-pig anti-VG1, 1:5000; rabbit anti-Homer1, 1:2000 and mouse anti-CytC, 1:3000; overnight at room temperature. After incubation, sections were washed in TBS, and sections were incubated overnight at 4°C in the mixtures of secondary antibodies (1) DyLight405-conjugated donkey-anti-goat (1:500, Jackson ImmunoResearch), Alexa Fluor 488-conjugated donkey-anti-rabbit (1:500, Life Technologies), and Alexa Fluor 647-conjugated donkey-anti-mouse (1:500, Jackson ImmunoResearch); (2) CF568-conjugated donkey-anti-rabbit (1:500, Jackson ImmunoResearch) and A647-conjugated donkey-anti-mouse (1:500, Jackson ImmunoResearch); or (3) DyLight405-conjugated donkey-anti-guinea-pig (1:500, Jackson ImmunoResearch), Alexa Fluor 488-conjugated donkey-anti-rabbit (1:500, Life Technologies) and Alexa Fluor 647-conjugated donkey-anti-mouse (1:500, Jackson ImmunoResearch). Secondary antibody incubation was followed by washes in TBS, PB, and hippocampi were cut out with scalpels under buffer, sections were dried on clean coverslips, and stored for maximum three weeks at room temperature in a dry environment before imaging. Imaging medium was mixed from 80 μl DPBS, 10 μl MEA solution, 10 μl 50% glucose solution, and 1 μl GLOX solution. Coverslips with the dried sections were mounted onto microscope slides with 25 μl of freshly prepared imaging medium, and sealed with nail polish. 3D direct-STORM (dSTORM) acquisition protocol was used as described before (Barna et al., 2016). The imaging setup was built around a Nikon Ti-E inverted microscope equipped with a Perfect Focus System, with an Andor iXon Ultra 897 EMCCD camera, a C2 confocal head, 405- and 488-nm lasers (Melles Griot 56RCS/S2780, Coherent Sapphire), and a high power 647-nm laser (300 mW, MPB Communications VFL-P-300-647). A high NA 100× oil immersion objective (Nikon CFI SR Apochromat TIRF 100× oil, 1.49 NA) was used for imaging. Emitted light was let through a 670/760-nm bandpass filter to reach the detector. We used the NIS-Elements AR 4.3 with N-STORM 3.4 software for acquisition and analysis. After selecting the region of interest, confocal image stacks were acquired containing 10 focal planes with 80 × 80 × 150 nm voxel size in *x*, *y*, and *z*, respectively. This was followed by bleaching the fluorophores in the STORM channels (561 and 647) with high intensity laser illumination, and running dSTORM acquisition using oblique (near-TIRF) illumination. Acquisition in the two channels was done in sequential mode. Confocal stacks were deconvolved (Huygens Professional), and, together with the STORM molecule lists, processed for further analysis in VividSTORM software (see below, Analysis and statistics). Imaging was performed with identical parameters (depth in the section, laser intensities, etc.) for all samples.

### Pre-embedding immunoelectron microscopy

For the immunogold labeling of CB1Rs, sections were washed in PB, treated with 0.5% sodium-borohydride for 13 min, and further washed in PB and TBS. After this, we incubated the sections in 1% HSA diluted in TBS. Then the sections were incubated for 48 h in rabbit anti-CB1R primary antibody diluted in TBS (1:500). After repeated washes in TBS, sections were treated with blocking solution (Gel-BS) containing 0.5% cold water fish skin gelatin (Aurion) and 0.5% HSA in TBS for 1 h. This was followed by 24-h incubation in 1.4 nm gold-conjugated goat anti-rabbit Fab fragment (1:200, NanoProbes) diluted in Gel-BS. After intensive washes in TBS and 0.1 M PB sections were treated with 2% GA in 0.1 M PB for 15 min to fix the gold particles into the tissue. This was followed by further washes in 0.1 M PB and enhancement conditioning solution (ECS, Aurion), gold particles were intensified using the silver enhancement solution (SE-EM, Aurion) for 40-60 min at room temperature. After subsequent washes, sections were treated with 0.5% osmium tetroxide in PB for 20 min on ice. Then sections were dehydrated in ascending ethanol series and acetonitrile, and embedded in epoxy resin (Durcupan, ACM, Fluka). During dehydration sections were treated with 1% uranyl acetate in 70% ethanol for 20 min. After polymerization, 60- or 200-nm-thick sections were cut using a Leica EM UC6 ultramicrotome, and picked up on formvar-coated single-slot copper grids; 60-nm-thick sections were examined using a Hitachi H-7100 EM and a side-mounted Veleta CCD camera (Olympus Soft Imaging Solutions), while 200-nm-thick sections were used for electron tomographic examinations.

### Serial-section EM of human samples

After washing the 60-µm-thick sections in 0.1 M PB, sections were treated with 0.5% osmium tetroxide in PB for 20 min on ice. Then sections were dehydrated in ascending ethanol series and acetonitrile, and embedded in epoxy resin (Durcupan, ACM, Fluka). During dehydration sections were treated with 1% uranyl acetate in 70% ethanol for 20 min. After polymerization, 60- or 200-nm-thick sections were cut using a Leica EM UC6 ultramicrotome, and picked up on formvar-coated single-slot copper grids; 60-nm-thick sections were examined using a Hitachi H-7100 EM (Tokyo, Japan) and a side-mounted Veleta CCD camera (Olympus Soft Imaging Solutions). Serial sections were examined, and mitochondria-containing glutamatergic boutons establishing asymmetric synapses were collected in the CA1 area, and reconstructed from long, serial sections. Measurement of synaptic areas and mitochondrial volumes were performed with Fiji/ImageJ software.

### Electron tomography

For the electron tomographic investigation, we used either 200-nm-thick sections from the hippocampal CA1 region from the anti-CB1R immunogold stained material (see above, Pre-embedding immunoelectron microscopy), or 100-nm-thick sections of unstained tissue from the dentate gyrus. In the latter case the sections were washed thoroughly in PB after postfixation (see above, Samples and tissue preparation), and treated with 0.5% osmium tetroxide in PB for 20 min on ice. Then sections were dehydrated in ascending ethanol series and acetonitrile, and embedded in epoxy resin (Durcupan). During dehydration sections were treated with 1% uranyl acetate in 70% ethanol for 20 min. Before electron tomography, serial sections on single-slot copper grids were photographed with a Hitachi H-7100 EM and a Veleta CCD camera. Serial sections were examined at lower magnification, and mitochondria-containing perisomatic synaptic boutons from the CA1 area, or mitochondria-containing glutamatergic synaptic boutons from the outer two-thirds of DG molecular layer were selected. At this magnification inner mitochondrial ultrastructure could not be observed on the TEM images from 100- and 200-nm-thick sections, securing the unbiasedness of sampling. In the CA1 experiments, CB1R immunogold staining was examined at higher magnification, and boutons were divided into CB1R+ and CB1R- (PV) categories. In the DG experiments, the boutons were reconstructed from the serial TEM image series, synapse sizes and mitochondrial volumes were measured. For each bouton, the section containing the largest mitochondrial cross section was chosen for electron tomography. After this, grids were put on drops of 10% HSA in TBS for 10 min, dipped in distilled water (DW), put on drops of 10 nm gold-conjugated Protein-A in DW (1:3), and washed in DW. Finally, we deposited 5-5 nm thick layers of carbon on both sides of the grids. Electron tomography was performed using a Tecnai T12 BioTwin EM equipped with a computer-controlled precision stage (CompuStage, FEI) equipped with a computer controlled precision stage (CompuStage, FEI) and an Eagle^™^ 2k CCD 4 megapixel TEM CCD camera (FEI). Acquisition was controlled via the Xplore3D software (FEI). Regions of interest were preilluminated for 6 min to minimize further shrinkage. Dual-axis tilt series were collected at two-degree increment steps between -65 and +65 degrees at 120-kV acceleration voltage and 23,000× magnification with −1.6- to −2-µm objective lens defocus. Reconstruction was performed using the weighted backprojection algorithm in the IMOD software package (RRID:SCR_003297; Kremer et al., 1996). Isotropic voxel size was 0.49 nm in the reconstructed volumes. After combining the reconstructed tomograms from the two axes, the nonlinear anisotropic diffusion (NAD) filtering algorithm was applied to the volumes. Segmentation of mitochondrial volumes has been performed on the virtual sections using the 3Dmod software, and measurements were done on the scaled 3D models.

### Methodological considerations

Electron tomography has become the primary method for studying biological structures in their native environment with the highest resolution. For these type of studies, it is critical to use tissue preparation techniques that most reliably preserve the physiologic state. Throughout the last decades, transcardial perfusion with aldehydes has become the gold standard method of EM tissue preparation when cryofixation is not an option (as in the case of intact brains). However, this technique is known to introduce some structural artefacts (decreased extracellular volume, decreased number of docked synaptic vesicles) into the sample when compared with rapid cryofixation (Korogod et al., 2015). Because of this, one needs to consider whether their results and the interpretation of these results are affected by these differences, or not.

In our case, there are two main reasons for being convinced, that our results are unaffected by this problem. Firstly, the parameters we set out to explore, namely the surface area and architecture of mitochondrial CMs, have been described to be unaffected by perfusion and aldehyde fixation when compared with rapid cryofixation and cryoelectron tomography (Nicastro et al., 2000; Perkins et al., 1998). Secondly, we always made comparisons within one system, and compared mitochondria that were sampled equally for each group from the same tissue volumes, meaning that they must have been affected identically, if anyhow. These arguments validate our approach, and enable us to draw biologically relevant conclusions from our data.

### Controls

**Figure 1.**
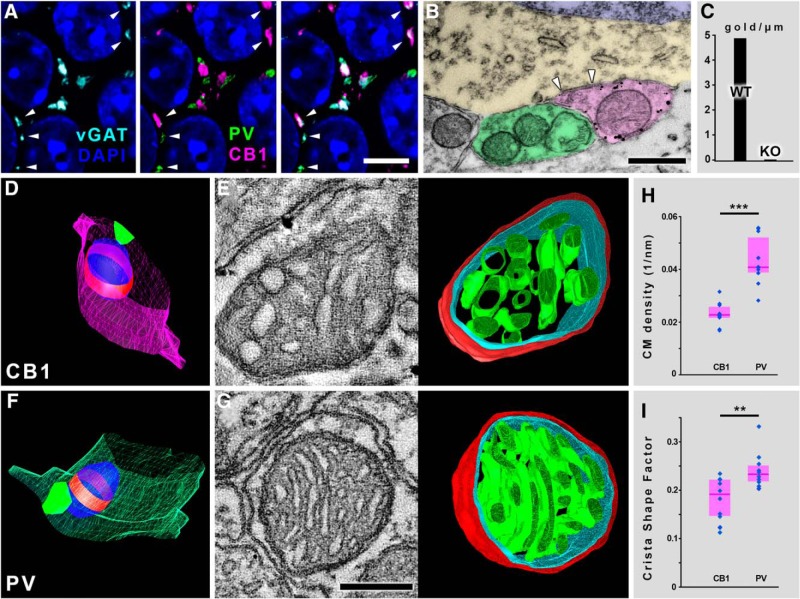
Electron tomography reveals robust ultrastructural differences between axonal mitochondria of fast-spiking and regular-spiking basket cells. ***A***, Confocal images show that perisomatic synaptic boutons are either PV+ or CB1R+ in the hippocampal CA1 region. Pyramidal cell nuclei are labeled with DAPI (blue), vGAT-immunoreactive puncta represent GABAergic vesicle pools (cyan), PV labeling is green and CB1R labeling is magenta, white arrowheads mark GABAergic release sites. ***B***, Transmission electron micrograph shows that CB1R-immunogold labeling reliably differentiates the two perisomatic bouton populations. Black granules are the silver-intensified CB1R-immunogold particles, white arrowheads mark a synapse. Pseudocolors: CB1R+ bouton, magenta; PV bouton, green; pyramidal cell cytoplasm, yellow; and nucleus, blue. ***C***, CB1R-immunogold labeling was absent in CB1R-KO animals (tested membrane length: 38 µm in two WT, and 74.8 µm in two CB1R-KO mice). ***D***, ***F***, Representative 3D models of serial EM-reconstructed segments from a synaptic CB1R+ bouton (***D***), and a synaptic PV bouton. Bouton membrane is magenta for CB1R bouton, green for PV bouton, synapses are vivid green, mitochondria are blue, and red belts represent those sections of the organelles that were reconstructed through electron tomography. ***E***, 1.5-nm-thick electron tomographic section and 3D model of the reconstructed mitochondrion from the bouton depicted in ***D*** (mitochondrial outer membrane is red, inner boundary membrane is cyan, CM is green). ***G***, 1.5-nm-thick electron tomographic section and 3D model of the reconstructed mitochondrion from the bouton depicted in F. ***H***, CM density is significantly higher in mitochondria of PV boutons than in those from CB1R+ boutons (Mann–Whitney *U* test, *p* = 0.0002, *n* = 20 mitochondria from two mice). ***I***, Cristae are significantly more lamellar in mitochondria of PV boutons than in those from CB1R+ boutons, as verified by the higher crista shape factor values (Mann–Whitney *U* test, *p* = 0.0090, *n* = 20 mitochondria from two mice). ***H***, ***I***, Blue dots represent values from individual mitochondria, magenta rectangles represent interquartile ranges, and deep-magenta lines mark median values. Scale bars: 6 µm (***A***), 500 nm (***B***), and 100 nm (***E***, ***G***). The 3D models in ***E***, ***G*** are not displayed on the same scale. See also Extended Data Figures 1-1, 1-2.

To validate the specificity and sensitivity of our labeling approach, we performed immunofluorescent reactions with two different CB1R antibodies in WT and CB1R-KO mice (Extended Data Fig. 1-1*A*,*B*). The two antibodies showed completely overlapping staining in the WT animals, but were totally absent in the KO animals (*n* = 2 WT and *n* = 2 KO mice). For the electron tomographic examinations, we performed immunogold labeling against CB1R (Fig. 1*B*), and quantified labeling density in WT and CB1R-KO animals (Fig. 1*C*). It was found to be 4.87 particle/µm on presynaptic CB1R+ boutons in WT, and 0.00 particle/µm in KO animals (tested along 38.6-µm membrane in two WT, and tested along 74.8-µm presynaptic bouton membrane in two KO animals). This would correspond to an average of 12 gold particles on a single EM section of a CB1R+ bouton in WT animals, reflecting the exceptionally high specificity and sensitivity of CB1R labeling, and confirming that this method can reliably distinguish between the two perisomatic bouton populations on serial EM images. To estimate the necessary preirradiaton of plastic embedded sections before electron tomography, we measured the dynamics of tissue shrinkage on 100- and 200-nm-thick sections. Shrinking happened mainly in the first 2 min of sample irradiation, and decreased to a negligible rate after 4-5 min (Extended Data Fig. 1-1*C*). After these measurements, we set a uniform preirradiation time of 6 min in our experiments.

10.1523/ENEURO.0390-17.2018.f1-1 Extended Data Figure 1-1 Staining with two anti-CB1R antibodies is overlapping in WT, but totally absent from CB1RKO mice; rate of tissue shrinkage due to electron beam irradiation. ***A***, Triple-color confocal images show CB1R staining in the hippocampal CA1 region of a WT mouse with a rabbit (green) and a goat (magenta) antibody. Cell nuclei are stained with DAPI (blue). ***B***, Staining with both antibodies are completely absent in CB1R-KO mice. Scale bar: 100 µm (upper panels) and 30 µm (lower panels). ***C***, Rates of tissue shrinkage on 100-nm-thick (left) and 200-nm-thick (right) sections under 15 min of electron beam irradiation. Black bars represent interquartile ranges, black dots mark median values. Download Figure 1-1, TIF file.

**Figure 2.**
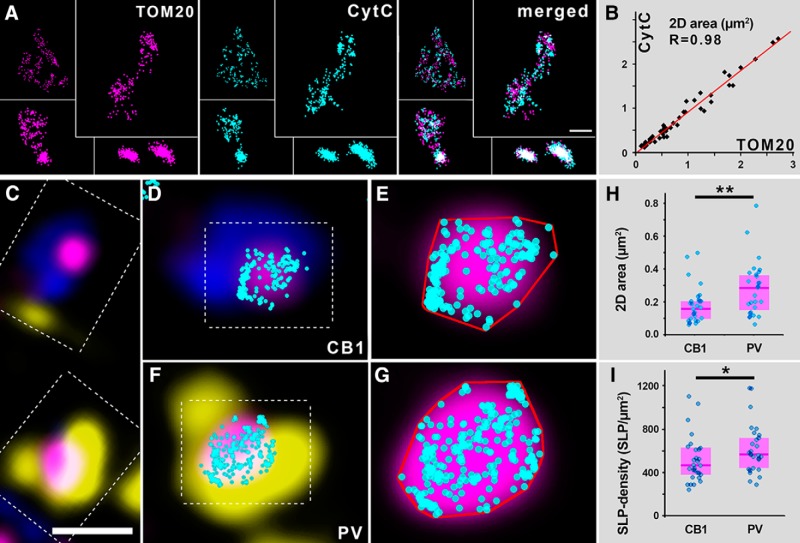
Axonal mitochondria of fast-spiking basket cells express higher levels of CytC than those of regular-spiking basket cells. ***A***, STORM super-resolution imaging confirms a near-complete overlap between TOM20 and CytC-labeled areas. ***B***, Scatterplot shows a strong correlation (*R* = 0.98, *n* = 48 mitochondria from two mice) between the measured areas in the two channels of individual mitochondria. Each dot corresponds to a single mitochondrion. ***C***, Confocal image shows a CB1R+ (blue) and a PV+ (yellow) perisomatic bouton, both containing a mitochondrion, labeled for CytC (magenta). ***D***, CB1R+ bouton enlarged from ***C*** with overlaid STORM CytC localization points (cyan). ***E***, Confocal and STORM image of mitochondrion enlarged from ***D***. ***F***, PV+ bouton enlarged from ***C*** with overlaid STORM CytC localization points. ***G***, Confocal and STORM image of mitochondrion enlarged from ***F***. Red lines in ***E***, ***G*** mark the 2D convex hulls generated around the localization points. ***H***, Mitochondria in the PV+ boutons are larger than those in the CB1R+ boutons, as the 2D areas of the convex hulls are significantly larger in the former group (Mann–Whitney *U* test, *p* = 0.0085, *n* = 57 mitochondria from two mice). ***I***, Mitochondria in the PV+ boutons contain CytC in a higher density than those in the CB1R+ boutons (Mann–Whitney *U* test, *p* = 0.0384, *n* = 57 mitochondria from two mice). Blue dots represent values from individual mitochondria, magenta rectangles represent interquartile ranges, and deep-magenta lines mark median values. Scale bars: 800 nm (***A***, upper-left mitochondrion), 400 nm (***A***, all others), 1 µm (***C***), 600 nm (***D***, ***F***), and 170 nm (***E***, ***G***).

10.1523/ENEURO.0390-17.2018.f1-2 Extended Data Figure 1-2 3D models of the reconstructed mitochondrial volumes from the hippocampal CA1 region. Top three rows show presynaptic mitochondria from regular spiking basket cell boutons, while the bottom three rows from fast-spiking basket-cell boutons. Mitochondrial outer membrane is red, inner boundary membrane is turquoise, CM is green. CM/MV, CM area/mitochondrial volume; CSF, crista shape factor. The models are not displayed on the same magnification. Download Figure 1-2, TIF file.

### Analysis and statistics

In the immunogold experiments the gold particles were considered to be membrane-associated, if they were not further away from the membrane than 40 nm, because the epitope (the C terminus of the CB1R) is located a few nanometers intracellularly, and the length of the primary and secondary IgG antibody molecules are ∼15 nm each.

During segmentation of tomographic volumes, z-scaling was calculated from the thickness difference of the reconstructed volume and the original section thickness, and applied to the 3D models to compensate for z-shrinkage. Mesh surface areas and volumes inside meshed objects were measured with the “imodinfo” program. To measure synaptic area in the serial TEM images, the length between the edges of anatomically defined synapses was measured on subsequent images of the sampled synapses, and the sum of the measured lengths from each synapse was multiplied by the section thickness.

In the confocal triple labeling experiments to visualize VG1-Homer-CytC signals, segmentation was performed separately for each channel to ensure unbiasedness.

STORM analysis was performed in the VividSTORM open source software (Barna et al., 2016). Localization points exceeding a given photon count were counted as specific super-resolution localization points (SLPs). Molecule lists were exported from NIS in txt format, and the three image planes of the ics-ids file pairs from the deconvolved confocal stacks matching the STORM volume were converted to the ome-tiff format using Fiji software. Confocal and corresponding STORM images were fitted in VividSTORM. Convex 2D hulls were automatically constructed around individual (not in fission or in fusion) mitochondria in both channels (568-TOM20 and 647-CytC), and organelle areas were correlated. During the examination of mitochondria in perisomatic boutons, the identity (CB1R+ vs PV+) of the boutons with mitochondria was determined in the confocal channels, and a convex 2D hull was constructed around the corresponding SLP cluster. The number of CytC SLPs was divided by the area of this convex hull to calculate the SLP density within mitochondria. During the examination of mitochondria in perforant pathway boutons, VG1+ boutons were identified with presynaptic mitochondria, and the size of the corresponding active zone was determined by 3D reconstruction of the Homer confocal signal. CytC SLP density was determined as described above.

When data populations did not have a Gaussian distribution according to the Shapiro-Wilks W test, we reported nonparametric statistical features (median, interquartile range), compared two independent groups with the Mann–Whitney *U* test, and Spearman’s correlation coefficients were calculated. When data populations showed normal distribution, correlations were assessed using linear regression fit to the data (*R* indicates Pearson’s correlation coefficient, Table 1). Statistical analysis was performed with the Statistica 13.1 package (RRID:SCR_014213, Dell), differences with *p* < 0.05 were considered significant throughout this study. Since data from different animals belonging to the same group were not statistically different in any case, they were pooled.

**Table 1.** Statistical table

Data structure	Comparison	Test
Unknown	Normality test	Shapiro–Wilks *W*
Not normal distribution	Two independent groups	Mann–Whitney *U*
Not normal distribution	Correlation	Spearman’s correlation
Normal distribution	Correlation	Pearson’s correlation

The applied tests, the n-numbers and exact p-values for each comparison are presented in the Results section and in the figure legends.

## Results

### Electron tomography reveals robust ultrastructural differences between presynaptic mitochondria of fast-spiking and regular-spiking GABAergic neurons

To test whether any correlation exists between synaptic performance and mitochondrial ultrastructure, we defined a low performance (LP) and a high performance (HP) group within hippocampal GABAergic as well as glutamatergic synaptic populations, based on the level of their activity. In the hippocampal CA1 area, the perisomatic GABAergic inputs of principal cells can be divided into two, morphologically and functionally well-characterized populations: the CB1R+, regular-spiking interneurons show low level of synaptic activity, whereas the PV+, fast-spiking interneurons are capable of performing exceptionally high synaptic activity. In fact, these GABAergic populations are the two well-known archetypes of the slow/modulatory, and the highly energized/fast “clockwork” type interneurons, respectively (Klausberger et al., 2005; Gulyás et al., 2006; Kann et al., 2014). The average *in vivo* firing frequency of the CB1R+ interneurons in the hippocampus is 2-10 Hz, whereas the PV+ interneurons can fire bursts up to 150 Hz during ripples (Klausberger et al., 2005; Lapray et al., 2012), making these two populations ideal candidates for LP and HP GABAergic boutons, respectively. Since the perisomatic synaptic boutons contacting pyramidal cells in the hippocampal CA1 region originate exclusively (99%) from these two cell types (Takács et al., 2015), labeling one of them is sufficient to reliably identify both types. To verify the exclusive and nonoverlapping contribution of the two populations with our antibodies as well, we performed immunofluorescent labeling of GABAergic varicosities for vGAT, the two bouton-populations for CB1R and PV, and cell nuclei with DAPI (Fig. 1*A*). We found, that 54.5% of all GABAergic boutons on pyramidal cell somata were PV+, 45% CB1R+, and only 0.5% were double-negative (*n* = 200 boutons in two mice), which numbers are in a very good agreement with previous data (Takács et al., 2015). For the electron tomographic examinations, we performed immunogold labeling against CB1R (Fig. 1*B*). Once the mitochondrion-containing boutons from both populations were sampled, the presence of a synapse was confirmed for each bouton (Fig. 1*D*,*F*). The sections containing the largest cross-sections of mitochondria were processed for dual-axis electron tomography and reconstruction, which revealed robust ultrastructural differences between these organelles, depending on which population they belonged to (Fig. 1*E*,*G*; Extended Data Fig 1-2). CM density (surface area of CM/mitochondrial volume) was significantly higher (by 79.7%) in mitochondria of PV boutons (median: 0.0408 nm^−1^, interquartile range: 0.0389–0.0519) than in those from CB1R+ boutons (median: 0.0227 nm^−1^, interquartile range: 0.0218–0.0256, Mann–Whitney *U* test, *p* = 0.0002, *n* = 20 mitochondria from two mice; Fig. 1*H*). When we multiplied these CM density values with the median volume of presynaptic mitochondria (119 × 10^6^ nm^3^ in PV+ and 73.4 × 10^6^ nm^3^ in CB1R+ boutons from Takács et al., 2015) we found a striking difference, showing that the average PV+ bouton possesses 2.9 times more mitochondrial CM surface than the average CB1R+ bouton. The organization of the cristae also differed, since these structures were significantly more lamellar in mitochondria of PV boutons than in those from CB1R+ boutons, as verified by the 22% higher crista shape factor values (surface area of CM/crista lumen volume, referred to as lamellarity throughout the paper), PV median: 0.233 nm^−1^, interquartile range: 0.218–0.251, CB1R median: 0.191 nm^−1^, interquartile range: 0.147–0.220, Mann–Whitney *U* test, *p* = 0.0090, *n* = 20 mitochondria from two mice; Fig. 1*I*). These results suggest a possible correlation between the ultrastructure of presynaptic mitochondria and the performance of the synapses.

### STORM super-resolution microscopy confirms higher mitochondrial CytC density in boutons of fast-spiking basket cells than in regular-spiking ones

To assess whether the observed ultrastructural differences are accompanied by any changes in respiratory chain protein expression, we turned to a combined STORM and CLSM method, and performed precise quantitative assessment of CytC levels in mitochondria. This protein is an indispensable element of the electron-transport chain, as it carries electrons to the CytC oxidase enzyme. CytC expression directly controls oxidative phosphorylation (Wilson et al., 2014), and its levels are indicative of mitochondrial performance and show good correlation with neuronal activity (Gulyás et al., 2006; Kann et al., 2014). To take advantage of the unprecedented 20-30 nm lateral localization precision and qunatitative nature of the STORM super-resolution technique (Huang et al., 2008) and delineate mitochondrial borders with high fidelity, we performed double labeling experiments. If the distribution of CytC is homogenous within individual mitochondria, then the CytC SLP clusters would designate the borders of these organelles in a reliable way. In this case, one could measure the sizes of these clusters and compare the SLP density values between different mitochondria. To test this, we stained for TOM20 (central component of the translocase of outer membrane receptor complex) and CytC (Fig. 2*A*). The measured sizes of individual mitochondria in the two STORM channels showed a very strong correlation (*R* = 0.98, *n* = 48 mitochondria from two mice, Fig. 2*B*), excluding the nonhomogenous sub-organelle distribution of CytC labeling. The median number of CytC SLPs labeling a single mitochondrion was 217 (interquartile range: 97–330). These results confirmed, that the CytC labeling and STORM imaging can be used to delineate the borders of mitochondria in a precise and reliable way. Next, we performed a multicolor immunofluorescent labeling for PV, CB1R and CytC, and imaged the samples using correlated confocal and STORM super-resolution microscopy (Fig. 2*C–G*). The CytC labeling was recorded with the STORM system as well. The STORM SLPs were overlayed the confocal images with the VividSTORM software (Fig. 2*D*,*F*). The analysis showed, that mitochondria in the PV+ boutons were larger than those in the CB1R+ boutons, as the 2D areas of the convex hulls, fitted around SLP-clusters, were 75.4% larger in the former group (median: 0.287 µm^2^, interquartile range: 0.158–0.368) than in the latter (median: 0.164 µm^2^, interquartile range: 0.098–0.203, Mann–Whitney *U* test, *p* = 0.0085, *n* = 57 mitochondria from two mice; Fig. 2*H*). More importantly we found, that mitochondria in the PV+ boutons contained CytC SLPs in a 22.7% higher density (median: 565.7 SLP/µm^2^, interquartile range: 434.8–725.7) than those in the CB1R+ boutons (median: 461.1 SLP/µm^2^, interquartile range: 378.8–612.0, Mann–Whitney *U* test, *p* = 0.0384, *n* = 57 mitochondria from two mice; Fig. 2*I*). These results confirm that the internal structural differences between mitochondria in the two bouton populations are accompanied by quantitative differences in molecular fingerprints as reflected by the levels of the respiratory chain protein CytC.

### Ultrastructural parameters of presynaptic mitochondria in glutamatergic boutons are coupled to synaptic strength

**Figure 3.**
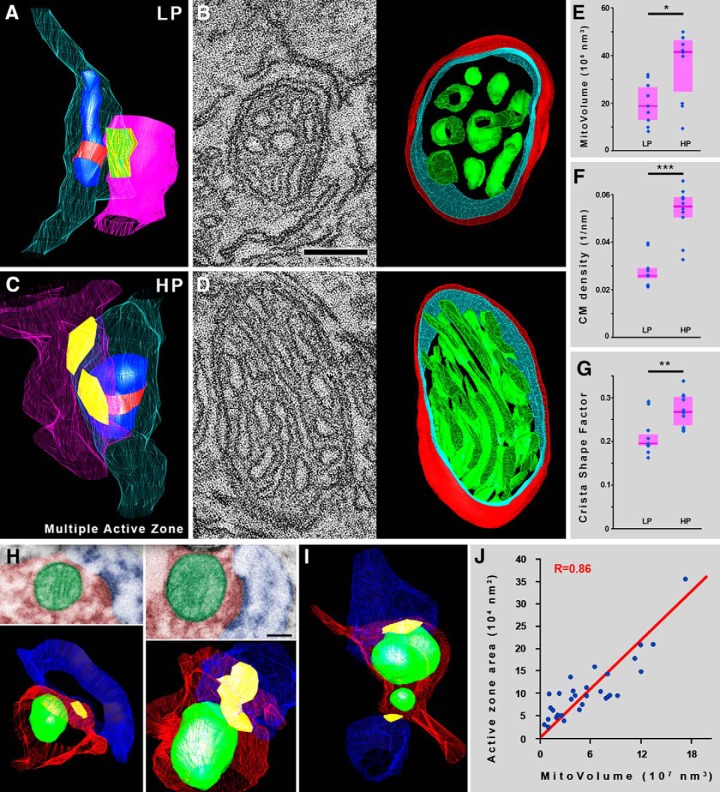
Mitochondrial ultrastructure is coupled to synaptic performance in a cell type-independent manner at glutamatergic synapses. ***A***, 3D model of a serial EM reconstructed segment of a LP glutamatergic bouton from the dentate gyrus. Bouton membranes are semitransparent cyan, postsynaptic profiles are semitransparent magenta, synapses are yellow, mitochondria are blue, and red belts mark those sections of the organelles that were reconstructed through electron tomography. ***B***, 1.5-nm-thick electron tomographic section and 3D model of the reconstructed mitochondrion from the bouton depicted in ***A*** (mitochondrial outer membrane is red, inner boundary membrane is cyan, CM is green). ***C***, 3D model of a serial EM reconstructed segment of a HP glutamatergic bouton from the dentate gyrus (colors same as in ***A***). ***D***, 1.5-nm-thick electron tomographic section and 3D model of the reconstructed mitochondrion from the bouton depicted in ***C***. ***E***, Mitochondria are significantly larger in HP boutons than in LP boutons (Mann–Whitney *U* test, *p* = 0.0199, *n* = 19 mitochondria from two mice). ***F***, CM density is significantly higher in mitochondria of HP boutons than in those from LP boutons (Mann–Whitney *U* test, *p* = 0.0009, *n* = 19 mitochondria from two mice). ***G***, Cristae are significantly more lamellar in mitochondria of HP boutons than in those from LP boutons, as verified by the higher crista shape factor values (Mann–Whitney *U* test, *p* = 0.0080, *n* = 19 mitochondria from two mice). ***H–J***, The volume of individual presynaptic mitochondria correlates with active zone area in the human hippocampus. ***H***, Transmission electron micrographs of two presynaptic mitochondria from human samples, and their 3D reconstructions from serial images. ***I***, 3D model of an axonal segment from human tissue, giving two synapses, each with an associated presynaptic mitochondrion. Axons are red, mitochondria green, spines blue, and active zones yellow. ***J***, The volume of presynaptic mitochondria shows a strong correlation with active zone area in human axons (*R* = 0.86, *p* < 0.0001, *n* = 31 mitochondria from two patients). ***E–G***, ***J***, Blue dots represent values from individual mitochondria, magenta rectangles represent interquartile ranges, deep-magenta lines mark median values. Scale bars: 110 nm (***B***, ***D***), 200 nm (***H***). The 3D models in ***B***, ***D***, ***H*** are not displayed on the same scale. See also Extended Data Figures 3-1, 3-2.

To confirm that the observed correlations between molecular and structural mitochondrial properties and synaptic features are independent of the cell types examined, we investigated glutamatergic synaptic boutons in the dentate gyrus. One of the best characterized structure-function correlations in neuroscience is that synaptic strength is strictly and faithfully linked to morphologic/ultrastructural parameters (Buchs and Muller, 1996; Desmond and Levy, 1988; Holtmaat and Svoboda, 2009; Bourne and Harris, 2012; Cheetham et al., 2014). The release probability of synapses scales linearly with the size of the active zone (Holderith et al., 2012), and long-term potentiation leads to an increase in the size of synaptic active zone, the number of perforated synapses and also in the number of boutons with multiple active zones (Geinisman, 2000; Popov et al., 2004). Based on this fundamental morpho-functional correlation, the strength of glutamatergic synapses can be determined purely by morphologic criteria in a very reliable way, enabling us to define a LP and a HP group within a specific glutamatergic synaptic bouton population. We randomly collected samples from the outer two-thirds of the dentate gyrus molecular layer, where the vast majority of glutamatergic contacts are made by perforant path axons (Amaral et al., 2007), and reconstructed synaptic boutons from serial EM sections together with the postsynaptic neuronal processes (Fig. 3*A*,*C*). The median active zone area was found to be 0.070 µm^2^ (0.042–0.123 µm^2^ interquartile range, *n* = 35 synaptic boutons in two mice; Extended Data Fig. 3-1*A*). About half of these boutons contained mitochondria, and the active zone area in this group did not differ significantly from the previous group, as it was found to be 0.093 µm^2^ (0.048–0.171 µm^2^ interquartile range, Mann–Whitney *U* test, *p* = 0.267; *n* = 19 synaptic boutons in two mice; Extended Data Fig. 3-1*A*). Based on these data and the strict correlation between synaptic ultrastructure and performance, we grouped the reconstructed mitochondria-containing varicosities as follows: boutons were considered to be LP, if they had a single, nonperforated active zone with an area <0.09 µm^2^, and boutons were considered to be HP, if they had multiple and/or perforated active zone(s) with an area larger than 0.09 µm^2^ (Extended Data Fig. 3-1*A*). We measured the mitochondrial volumes in the 3D models, and found that presynaptic mitochondria in HP boutons were significantly larger than those in LP boutons (HP median: 41.8 × 10^6^ nm^3^, 25 × 10^6^ to 46.5 × 10^6^ interquartile range, LP median: 18.9 × 10^6^ nm^3^, 13 × 10^6^ to 27.6 × 10^6^ interquartile range, 121.6% difference, Mann–Whitney *U* test, *p* = 0.0199, *n* = 19 boutons from two mice; Fig. 3*E*). The sections containing the largest cross-sections of presynaptic mitochondria of these boutons were processed for dual-axis electron tomography and reconstruction, which revealed robust ultrastructural differences between these organelles, depending on which population they belonged to (Fig. 3*B*,*D*; Extended Data Fig. 3-2). CM density (surface area of CM/mitochondrial volume) was significantly higher (by 110.1%) in mitochondria of HP boutons (median: 0.0552 nm^−1^, interquartile range: 0.0509–0.0587) than in those from LP boutons (median: 0.0262 nm^−1^, interquartile range: 0.0258–0.0290, Mann–Whitney *U* test, *p* = 0.0009, *n* = 19 mitochondria from two mice; Fig. 3*F*). We then multiplied these CM density values with the median volume of presynaptic mitochondria (41.8 × 10^6^ nm^3^ in HP and 18.9 × 10^6^ nm^3^ in LP boutons), and found a striking difference, showing that the average HP bouton possesses 4.67 times more mitochondrial CM surface than the average LP bouton. The organization of the cristae also differed significantly, since these structures were more lamellar in mitochondria of HP boutons than in those from LP boutons, as verified by the 37.8% higher crista shape factor values (surface area of CM/crista lumen volume) in the former group (median: 0.268, interquartile range: 0.237–0.301), than in the latter (median: 0.195, interquartile range: 0.190–0.219, Mann–Whitney *U* test, *p* = 0.0080, *n* = 19 mitochondria from two mice; Fig. 3*G*). To verify the robustness of our results, we pooled data from HP and LP boutons, and examined whether the different ultrastructural features correlated with the size of the synaptic active zone. We found that the volume, CM density and crista shape factor of presynaptic mitochondria are all strongly and significantly correlated with synapse size (*R* = 0.71, *p* = 0.0005; *R* = 0.77, *p* = 0.0001; and *R* = 0.65, *p* = 0.0026, respectively; Pearson correlation, *n* = 19 mitochondria from two mice; Extended Data Fig. 3-1*B*). We also tested our findings on postmortem human tissue. We collected a random sample of mitochondria-containing presynaptic glutamatergic axon segments from the stratum radiatum of the hippocampal CA1 region, reconstructed them from serial sections (Fig. 3*H*), and found a strong and significant correlation between the active zone area and the volume of individual presynaptic mitochondria (*R* = 0.86, *p* < 0.0001, *n* = 31 mitochondria from two patients; Fig. 3*I*,*J*), confirming the results from mouse studies. In summary, our results reveal for the first time that the ultrastructure of presynaptic mitochondria is coupled to synaptic performance in the brain, in a cell type-independent manner.

10.1523/ENEURO.0390-17.2018.f3-1 Extended Data Figure 3-1 Distribution of active zone sizes of the glutamatergic boutons in the tomographic and STORM measurements, and correlations of performance-determining mitochondrial features with synapse size. ***A***, Active zone area distribution of the glutamatergic boutons examined with serial EM and electron tomography. ***B***, The volume, CM density, and crista shape factor of presynaptic mitochondria are all strongly correlated to synapse size (*R* = 0.71, *p* = 0.0005; *R* = 0.77, *p* = 0.0001; and *R* = 0.65, *p* = 0.0026, respectively). Blue dots represent values from individual mitochondria, regression lines are red. ***C***, Active zone area distribution of the glutamatergic boutons examined with CLSM and STORM super-resolution microscopy. All syn., all examined synaptic boutons; Mito. syn., synaptic boutons with mitochondria; LP syn., LP synaptic boutons; HP syn., HP synaptic boutons. Blue dots represent values from individual boutons, magenta rectangles represent interquartile ranges, deep-magenta lines mark median values. Red lines show values dividing HP and LP synaptic boutons. D The CytC-labeled volume and SLP density are both strongly correlated to synapse size (*R* = 0.77, *p* < 0.00001; *R* = 0.60, *p* = 0.00002, respectively). Blue dots represent values from individual mitochondria, regression lines are red. Download Figure 3-1, TIF file.

10.1523/ENEURO.0390-17.2018.f3-2 Extended Data Figure 3-2 3D models of the reconstructed mitochondrial volumes from the dentate gyrus. Top three rows show presynaptic mitochondria from LP glutamatergic boutons, while the bottom three rows from HP ones. Mitochondrial outer membrane is red, inner boundary membrane is turquoise, CM is green. CM/MV, CM area/mitochondrial volume; CSF, crista shape factor. The models are not displayed on the same magnification. Download Figure 3-2, TIF file.

### CytC density in presynaptic mitochondria of glutamatergic boutons scales with synaptic strength

**Figure 4.**
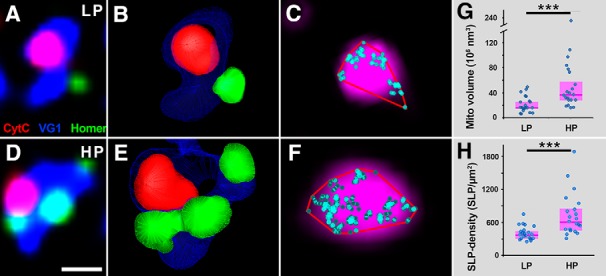
CytC content of presynaptic mitochondria scales with synaptic performance at glutamatergic synapses. ***A***, Confocal image shows a glutamatergic bouton (VG1, blue), containing a mitochondrion (Cyt-C, red), and its synapse (Homer1a, green). ***B***, The 3D model of the bouton from ***A*** was reconstructed from the confocal stack. ***C***, Confocal and STORM image of mitochondrion enlarged from the same bouton (Cyt-C confocal in magenta, Cyt-C localization points in cyan, 2D convex hull in red). ***D–F***, Another bouton depicted as in ***A–C***. Mitochondria-containing synaptic boutons were divided into LP and HP groups, based on the corresponding Homer volumes. Bouton in ***A–C*** represents a LP, bouton in ***D–F*** represents a HP bouton. ***G***, Mitochondria in the HP boutons are larger than those in the LP boutons, as the CytC-volumes are significantly larger in the former group (Mann–Whitney *U* test, *p* = 0.00032, *n* = 42 mitochondria from two mice). ***H***, Mitochondria in the HP boutons contain CytC in a higher density than those in the LP boutons (Mann–Whitney *U* test, *p* = 0.00016, *n* = 42 mitochondria from two mice). Blue dots represent values from individual mitochondria, magenta rectangles represent interquartile ranges, deep-magenta lines mark median values. Scale bars: 800 nm (***A***, ***D***), 200 nm (***C***, ***F***). The 3D models on ***B***, ***E*** are not displayed on the same scale. See also Extended Data Figure 3-1.

To examine whether the observed cell type-independent coupling between mitochondrial ultrastructure and synaptic performance is accompanied also by changes in respiratory chain protein expression levels, we tested the density of CytC labeling in mitochondria of LP and HP glutamatergic synaptic boutons, using correlated confocal and STORM super-resolution microscopy, taking advantage of the near-molecular resolution capability of this method. We labeled glutamategic boutons against VG1, mitochondria with CytC, and postsynaptic densities with Homer1 (Fig. 4*A*,*D*). We sampled mitochondria-containing synaptic boutons from the outer two-thirds of DG molecular layer, and reconstructed them from the deconvolved confocal stacks (Fig. 4*B*,*E*). The volumes of Homer labeling belonging to each bouton were measured on the 3D models, and the median value was found to be 0.0265 µm^3^ (0.011–0.0518 interquartile range; Extended Data Fig. 3-1*C*). These values correspond to the size of the active zone in each bouton, meaning that this distribution indirectly reflects the output performance distribution of the examined boutons. Based on these data, we split the population at the median value, and sorted boutons with a lower Homer volume into the LP group, and boutons with higher values into the HP group (Extended Data Fig. 3-1*C*). We found that presynaptic mitochondria in HP boutons were significantly larger than those in LP boutons (HP median: 0.0365 µm^3^, 0.0282–0.0578 interquartile range, LP median: 0.0162 µm^3^, 0.0150–0.0265 interquartile range, 128.8% difference, Mann–Whitney *U* test, *p* = 0.0003, *n* = 42 boutons from two mice; Fig. 4*G*), and this observed difference is congruent with our serial EM data (128.8% vs 121.6% difference). The CytC labeling was recorded not only in the confocal channel, but also in the correlated STORM modality. The STORM SLPs were overlayed the confocal images (Fig. 4*C*,*F*). We found that the CytC SLP density was 61% higher in mitochondria of HP boutons than in those of LP boutons (HP median: 598.5 SLP/µm^2^, 455.6–822 interquartile range, LP median: 370.8, 296.3–440.7 interquartile range, Mann–Whitney *U* test, *p* = 0.0001, *n* = 42 boutons from two mice; Fig. 4*H*). To verify the strength of our results, we pooled data from HP and LP boutons, and examined whether the investigated features correlated with the size of the synaptic active zone. We found, that the CytC-labeled volume and SLP density are both strongly correlated to synapse size (*R* = 0.77, *p* < 0.00001; *R* = 0.60, *p* = 0.00002, respectively; Spearman’s correlation, *n* = 42 mitochondria from two mice; Extended Data Fig. 3-1*D*). Taken all these together, our results confirm that not only the mitochondrial ultrastructure is coupled to synaptic performance in a cell type-independent manner, but also the expression levels of the respiratory chain protein CytC.

## Discussion

### Mitochondrial and synaptic performance are coupled via ultrastructure

The first observations suggesting a connection between mitochondrial crista structure and function were obtained on *ex vivo* isolated mitochondria (Hackenbrock, 1966). Although those specific results have not been verified under physiologic conditions, causal correlation between mitochondrial ultrastructure and output performance in non-neuronal cells has been confirmed in recent studies (Cogliati et al., 2016). The amount and density of CM as well as the lamellarity of cristae directly determine respiratory performance and efficiency (Else et al., 2004; Cogliati et al., 2013), and these ultrastructural features were also shown to influence performance at the level of the complete organism (Strohm and Daniels, 2003). On the other hand, aberrant morphology, dilatation or loss of mitochondrial cristae have been observed in a variety of pathologic conditions (Daum et al., 2013; Choi et al., 2014), while genetic manipulations ameliorating damaged crista structure have been shown to restore normal crista density/lamellarity and respiratory chain activity (Civiletto et al., 2015). Furthermore, modeling studies also confirmed, that CM density and lamellarity are key determinants of mitochondrial electrochemical potential and ATP-generating capacity (Song et al., 2013). There are several points explaining the correlation between mitochondrial ultrastructure and performance: larger CM surface can accommodate a higher amount of respiratory chain and ATP-synthase proteins, leading to a higher respiratory and ATP-generating capacity, while more lamellar cristae structure is beneficial for respiratory chain supercomplex assembly, resulting in a higher respiratory efficiency (Cogliati et al., 2013). Furthermore, more lamellar cristae, independently from changes in respiratory efficacy, result in an increased proton motive force, leading to higher ATP-producing capacity (Song et al., 2013). Finally, ATP-synthase molecules tend to be enriched and dimerize at curved cristae rims, making the dense and tight packing of these membranes more effective to produce ATP (Davies et al., 2011).

Mitochondria are critically important for proper synaptic function, due to their central role in ATP production, Ca^2+^ regulation, and other major signaling mechanisms. In particular, presynaptic function has been shown to rely directly on activity-driven ATP synthesis (Rangaraju et al., 2014). The demand for mitochondrial function is reasonably coupled to neuronal activity (Gulyás et al., 2006; Kann et al., 2014) and also directly correlated with synaptic strength (Verstreken et al., 2005; Ivannikov et al., 2013; Sun et al., 2013; Smith et al., 2016). Our results demonstrate that increased synaptic performance is coupled to higher organelle volume, crista density, crista lamellarity and CytC levels at axonal release sites, suggesting that ultrastructure and molecular composition of presynaptic mitochondria are associated with synaptic performance.

### Performance coupling between mitochondria and synapses suggests a possible role for the activity dependent ultrastructural remodeling of mitochondria in neuroplasticity

Neuronal mitostasis, the maintenance of an appropriately distributed pool of healthy mitochondria, is fundamental for proper neuronal function (for review, see Misgeld and Schwarz, 2017). Our observations verified the presence of a cell type-independent coupling between presynaptic mitochondrial ultrastructure and synaptic strength. This coupling, responsible for local demand matching, could be achieved either by a coordinated long-range retrograde/anterograde axonal trafficking of mitochondria, or by local plasticity mechanisms, that lead to adaptation at the level of single organelles.

During synaptic plasticity, the energetic demand of a potentiated synapse will increase. In case of the first mechanism, organelle trafficking, the mitochondrion from the potentiated bouton would need to be withdrawn, transported away, possibly back to the soma, and another, stronger mitochondrion would be sent out to replace it. The coordination of this process, together with the evident gap in energy supply during the change is more than problematic. On the other hand, the large number of papers dealing with mitochondrial motility and redistribution performed mainly *in vitro* experiments, however, recent *in vivo* two-photon imaging studies suggest that mitochondrial motility is way much smaller, and also quite independent from neuronal activity (Lewis et al., 2016; Smit-Rigter et al., 2016). This suggests that this mechanism alone is not sufficient to account for local demand matching. The other possible mechanism is locally regulated ultrastructural plasticity. In fact, in non-neuronal tissues, an overwhelming body of evidence confirms that dynamic ultrastructural remodeling of mitochondria takes place as a response to altered energetic demand (Cogliati et al., 2016).

Mitochondrial inner membrane architecture has been shown to undergo substantial remodeling after exercise, under hypoxic conditions or during starvation (Hambrecht et al., 1997; Gomes et al., 2011; Perkins et al., 2012; Nielsen et al., 2017), suggesting that the local demand for mitochondrial performance is communicated to these organelles, prompting their activity-dependent ultrastructural plasticity. The common response to an increased energetic demand was an increased CM density and lamellarity in all of these cases. The idea of mitochondrial ultrastructural adaptation is also supported by a study, confirming that crista remodeling can happen on the timescale of minutes (Dikov and Bereiter-Hahn, 2013). In recent years, various mechanisms have been described to regulate mitochondrial ultrastructure, such as mitochondrial dynamin like GTPase (OPA1), mitochondrial contact site and cristae organizing system (MICOS), ATP-synthase dimerization and the inner-membrane protein Pantagruelian mitochondrion I (Gomes et al., 2011; Harner et al., 2011; Neupert, 2012; Macchi et al., 2013; Hahn et al., 2016). Substrate dependent changes in OPA1 oligomer levels (Patten et al., 2014; Sood et al., 2014), or activity-dependent ATP-synthase clustering (Jimenez et al., 2014) are known to contribute to mitochondrial ultrastructural plasticity. Synaptic mitochondria possess a specific proteome (Völgyi et al., 2015), and local axonal protein synthesis has also been shown to be required for the maintenance of mitochondrial function (Hillefors et al., 2007; Aschrafi et al., 2008; Gale et al., 2017). Thus, it is very likely that local presynaptic signaling mechanisms regulate the expression of mitochondrial proteins in axons, thereby controlling mitochondrial ultrastructure and performance.

All these findings, together with our results, suggest that the primary way of adjusting mitochondrial performance to the actual demand at synapses could be the activity-dependent ultrastructural plasticity of these organelles. Furthermore, our results, confirming a cell type-independent coupling between synaptic performance and mitochondrial ultrastructure, indicate that these changes in mitochondrial ultrastructure and molecular fingerprints could contribute to neuroplasticity at the level of individual synapses.

## Synthesis

Reviewing Editor: Pablo Castillo, Albert Einstein College of Medicine Decisions are customarily a result of the Reviewing Editor and the peer reviewers coming together and discussing their recommendations until a consensus is reached. When revisions are invited, a fact-based synthesis statement explaining their decision and outlining what is needed to prepare a revision will be listed below. The following reviewer(s) agreed to reveal their identity: Thomas Blanpied

Your manuscript has been evaluated by two experts in the field. They both agree on the quality and significance of your results. They understand your study provides convincing evidence for a link between presynaptic mitochondria structure and synaptic activity . As you will see below, they also raised a number of points that you should properly address before your paper can be considered for publication.

---------------------------------------------

REVIEWER 1

In the present study "Mitochondrial Ultrastructure Is Coupled to Synaptic Performance at

Axonal Release Sites" the authors analyzed presynaptic mitochondria of hippocampal cells of the CA1 area and of the dentate gyrus. The study revealed that mitochondria show significant differences in structure, size and protein levels dependent on the activity of the synapse but independent on the cell-type. "High performance" synaptic boutons bear mitochondria with lamellar cristae, "low performance" synaptic boutons, however, bear mitochondria with cristae with a very different, ring-shaped structure. In addition, the mitochondria of highly active synapses contained a higher number of cristae than the mitochondria in less active synapses. Further data support the suggestion of a higher amount of respiratory chain complexes in mitochondria of highly active synapses. This difference appears to reflect the higher energy demand of the "high performance" synaptic boutons. The connection between mitochondrial ultrastructure and cellular function is an interesting aspect that has been observed in a number of organisms, tissues and cells and in this respect is not novel. But in neuronal cells this is an important, so far not investigated topic. In this context, the authors provide convincing evidence using elaborate, extensive cutting edge microscopy. In particular, the analysis by electron tomography is of excellent quality.

Major Points:

- The results obtained with human tissue fit into the story. However, in the context of this study it would be necessary to analyze the mitochondrial ultrastructure. A further problem is whether the number of n=16 mitochondria is statistically significant. Therefore, I would suggest to omit these data.

- The Discussion section has to be revised:

o First paragraph:

Some of the conclusions in this paragraph are far-fetched and possibly misleading.

Increase in crista tightness does not correctly explain what the authors observe here. The authors show that mitochondria of "low performance" synaptic boutons bear ring-shaped cristae whereas mitochondria of "high performance" synaptic boutons show lamellar cristae. These are significantly different structures. Increased crista tightness apparently means that the inner distance between the two membranes is lower. This distance is higher and constant in ring-shaped cristae due to the circular cross section. Lamellar cristae of highly active synapses, however, show a small distance due to the highly bent sheet-like structure. However, it is still a matter of debate whether this "tightness" has any effect of the proton motive force. Furthermore, the studies of Kuhlbrandt's and other labs show that dimeric ATP-synthase is present at bent membrane. Thus, dimeric ATP-synthase is highly enriched at the rims of a lamellar crista. The large planar area of a lamellar crista provides abundant space for respiratory chain complexes. This is indeed correct. However, dimeric ATP-synthase may also be present in ring-shaped cristae bending the membrane to a lower degree. In addition, dimerization of the ATP-synthase is independent on its enzymatic function.

I suggest to skip the term crista tightness completely. However, the authors should explain in more detail that ring-shaped cristae are present in one kind of boutons whereas lamellar cristae are present in the other. This might indicate differences in the function of mitochondria for example in PV- and CB1R-positive cells. Changes in mitochondrial ultrastructure upon changes in cellular function have been observed before.

Since the paragraph starting from lane 665 (whilst tighter cristae...) - 670 (more effective to produce ATP) is not relevant for the conclusions and is too speculative, I suggest that it should be omitted.

o Second paragraph:

The heading and discussion has to be changed. "Ultrastructural remodelling" suggests that "low performance" synaptic boutons can be converted in "high performance" synaptic boutons and vice versa. There is no evidence and no reason for this suggestion.

The statement "the local demand for mitochondrial performance is communicated to these organelles, prompting their activity-dependent ultrastructural plasticity" does not reflect the situation. There are different types of cells with different mitochondria with different ATP production and possibly other functions. There is no "remodeling" or "ultrastructural plasticity". The differences of the observed ultrastructures are probably the result of unknown signaling processes that sense the need for more or less respiratory complexes and communicate this to nuclear and mitochondrial transcription. Lamellar cristae provide much more space than tubular cristae and are therefore formed predominantly in these cells.

o Patten et al. did clearly show that the steady state level of Opa1 did not vary upon starvation, but its oligomeric state. This has to be changed in the discussion!

Minor Points:

- The authors use the terms "ultrastructure", "sub-organelle ultrastructure" and "nano-scale architecture" that have an identical meaning. This is confusing. The usual term in the literature is "ultrastructure".

- The abbreviations of buffers and their compositions should be given.

- Error: Line 553 Fig. 3-2. B should be Fig. 3-1. B.

- It would be appropriate to show directly that the mitochondrial reconstructions provided in Fig 3 are derived from boutons with a non-perforated active zone or from boutons with a multiple-perforated active zone.

- The completely different scales in Figure 3E and 4G are confusing. I recommend to keep it use the same scale in both figures.

REVIEWER 2

This paper is among the many that have recently begun to re-address an important overarching topic - whether/how are synaptic transmission and plasticity reliant upon mitochondrial function by means of regulation of mitochondrial shape and dynamics. This had begun to be studied decades ago, but now with new and improved structural and functional assays, the field is experiencing a resurgence. In line with this, these authors use a lovely combination of transmission EM, confocal, and localization-based microscopy to study their hypothesis that presynaptic mitochondrial ultrastructure controls mitochondrial function in order to meet synaptic needs. This manuscript tests a couple predictions of this hypothesis, specifically whether mitochondrial structure is correlated with mitochondrial function (or rather, structural readouts with inferred functional implications) and synaptic function. Furthermore, the authors test whether this is synapse specific by studying three different synapses. They also test whether this is cell-type specific by morphometrically subcategorizing synapses within one category (PP-DG) into high and low performance.

The authors' overarching conclusion is that "not only the mitochondrial nano-architecture is coupled to synaptic performance in a cell-type independent manner, but also the expression of the levels of the respiratory chain protein cytochrome c". Furthermore, the authors contextualize their findings with the literature and state "the primary way of adjusting mitochondrial performance to the actual demand at synapses could be the activity-dependent ultrastructural plasticity of these organelles...these changes in mitochondrial nano-architecture and molecular fingerprints could contribute to neuroplasticity at the level of individual synapses". This manuscript is an incomplete study of the large stated hypothesis. However, many of the individual conclusions within the manuscript are supported by the data presented, while only a small subset are not. Nevertheless, a couple of small additions would help clarify the conclusions presented.

- In 1 H - CM density is higher in PV. But, crista width seems to be greater in CB1R. Does this have any putative functional relevance? Refer to HL Smith et al, eLife 2016.

- It would be helpful if the authors could explain why the CM density is an appropriate measure here. Cogliati et al, 2013 suggest blebbier cristae appear approaching the "apoptotic" spectrum and thus these mitos have less respiratory functional capacity.

- In addition to CM density and crista shape factor, the authors could also measure cristae width, which was recently (HL Smith et al, eLife 2016) correlated with an increase in presynaptic function following TBS-LTP. Is cristae width also greater in HP/PV compared to LP/CB1R boutons?

- Comparing even a few HP and LP boutons from the same axon (similar to HL Smith et al., eLife 2016, and others) would strengthen the conclusion. Is there a way to do this in the existing data?

- The sentence "These results suggest a strong correlation between the ultrastructure of presynaptic mitochondria and the performance of synapses" is perhaps overstated. All the authors can conclude thus far is that mitochondria within two different populations of basket cell boutons are ultrastructurally different. Perhaps "presumed performance" or some such minor qualifier?

- For Fig 2, authors should clarify that they mean that the BORDERS of the Tom20 and CytC are overlapping, not that the labeled protein points overlap. And from a technical standpoint, it is not clear (perhaps I missed a detail in the methods) whether the points shown for the staining are those that passed some set of criteria to help define the mitochondria or if that is all the localizations from the staining. Are the points shown after processing or "raw data"?

- The authors state that "the average HP bouton utilizes 4.67 times more mitochondrial crista membrane surface than the average LP bouton". Without a functional assay, the use of the term "utilizes" seems inappropriate.

- Was the image quality high enough in the human sample to analyze CM density or crista shape? Those values would be useful to include.

Figures-

- It would be helpful to have text in the panel stating which bouton type we're observing. This comment is applicable to the first panels of each of the 4 figures. Similarly, I'm not a fan of the gray background in the graphs, which decreases contrast and readability.

- Axis labels and units are missing in Fig. 2B.

- Figure 1A says CitC instead of CytC.

## Author Response

Synthesis of Reviews:

Computational Neuroscience Model Code Accessibility Comments for Author (Required):

The authors used their lab's in-house software called VividSTORM. This is not new, though it is not clear whether new toolboxes or other aspects are new here.

VividSTORM is continuously developed, however, in this study we did not use any new features.

Significance Statement Comments for Author (Required):

n/a

Comments on the Visual Abstract for Author (Required):

The Abstract reflects the conclusion but should be adapted according to the comments regarding the term “crista tightness”.

The Visual Abstract has been corrected accordingly.

Synthesis Statement for Author (Required):

Your manuscript has been evaluated by two experts in the field. They both agree on the quality and significance of your results. They understand your study provides convincing evidence for a link between presynaptic mitochondria structure and synaptic activity. As you will see below, they also raised a number of points that you should properly address before your paper can be considered for publication.

Response to Reviewer #1:

REVIEWER 1

In the present study “Mitochondrial Ultrastructure Is Coupled to Synaptic Performance at

Axonal Release Sites” the authors analyzed presynaptic mitochondria of hippocampal cells of the CA1 area and of the dentate gyrus. The study revealed that mitochondria show significant differences in structure, size and protein levels dependent on the activity of the synapse but independent on the cell-type. “High performance” synaptic boutons bear mitochondria with lamellar cristae, “low performance” synaptic boutons, however, bear mitochondria with cristae with a very different, ring-shaped structure. In addition, the mitochondria of highly active synapses contained a higher number of cristae than the mitochondria in less active synapses. Further data support the suggestion of a higher amount of respiratory chain complexes in mitochondria of highly active synapses. This difference appears to reflect the higher energy demand of the “high performance” synaptic boutons. The connection between mitochondrial ultrastructure and cellular function is an interesting aspect that has been observed in a number of organisms, tissues and cells and in this respect is not novel. But in neuronal cells this is an important, so far not investigated topic. In this context, the authors provide convincing evidence using elaborate, extensive cutting edge microscopy. In particular, the analysis by electron tomography is of excellent quality.

Major Points:

- The results obtained with human tissue fit into the story. However, in the context of this study it would be necessary to analyze the mitochondrial ultrastructure. A further problem is whether the number of n=16 mitochondria is statistically significant. Therefore, I would suggest to omit these data.

We agree with the Reviewer that inclusion of human data is in line with the scope of the work and that measurements of mitochondrial ultrastructure in human tissue would further strengthen our findings. However, in spite of the short post-mortem time before fixation (4-5 hours) concerning the samples used for analysis, the preservation of post-mortem human tissues does not allow to reliably measure parameters that are indicative of mitochondrial performance beyond the assessment of mitochondrial volume and active zone area on serial electron microscopic images. Since the volume of mitochondria is strongly associated with other parameters used as reliable measures of mitochondrial performance in the study - as demonstrated in the case of mouse tissues - we believe that the human data are valuable additions to this work. Involvement of these data provides further support for the relevance of our findings, since only very few high-resolution imaging data obtained in animal studies are validated by using human samples. Furthermore, the present data provide promising opportunities to study changes in mitochondrial anatomical features in common human neurological diseases that are assumed to be influenced by changes in synaptic function and performance (Choi et al., 2014; Daum et al., 2013, Brandt et al., Elife. 2017 Jul 12;6. pii: e24662.). However, we agree with the reviewer that drawing conclusions based on n=16 mitochondria from a single human brain might raise issues regarding statistical power (although the correlation between the active zone area and the volume of individual presynaptic mitochondria is significant even at this n-number: p=0.0003, Pearson's correlation). To this end, we obtained tissue samples from another patient, repeated the measurement and included these data to the manuscript. The figure, the Methods and the Results have been corrected accordingly. We now have n=31 mitochondria from two patients, with a clear correlation between mitochondrial volume and synaptic active zone area.

- The Discussion section has to be revised:

o First paragraph:

Some of the conclusions in this paragraph are far-fetched and possibly misleading.

Increase in crista tightness does not correctly explain what the authors observe here. The authors show that mitochondria of “low performance” synaptic boutons bear ring-shaped cristae whereas mitochondria of “high performance” synaptic boutons show lamellar cristae. These are significantly different structures. Increased crista tightness apparently means that the inner distance between the two membranes is lower. This distance is higher and constant in ring-shaped cristae due to the circular cross section. Lamellar cristae of highly active synapses, however, show a small distance due to the highly bent sheet-like structure. However, it is still a matter of debate whether this “tightness” has any effect of the proton motive force. Furthermore, the studies of KÃ¼hlbrandt's and other labs show that dimeric ATP-synthase is present at bent membrane. Thus, dimeric ATP-synthase is highly enriched at the rims of a lamellar crista. The large planar area of a lamellar crista provides abundant space for respiratory chain complexes. This is indeed correct. However, dimeric ATP-synthase may also be present in ring-shaped cristae bending the membrane to a lower degree. In addition, dimerization of the ATP-synthase is independent on its enzymatic function.

I suggest to skip the term crista tightness completely. However, the authors should explain in more detail that ring-shaped cristae are present in one kind of boutons whereas lamellar cristae are present in the other. This might indicate differences in the function of mitochondria for example in PV- and CB1R-positive cells. Changes in mitochondrial ultrastructure upon changes in cellular function have been observed before.

We would like to thank the Reviewer for the comments. As suggested, we skip the term “crista tightness” and instead, we use the more common term “lamellar” throughout the paper. We use this term to describe the “flatness” of cristae, measured by “crista shape factor” (surface area of crista membrane/crista lumen volume). The latest studies in the literature and our observations also suggest that there is a continuous spectrum of mitochondrial ultrastructure, ranging from mitochondria with lower crista membrane density, less lamellar cristae and lower respiratory activity to mitochondria possessing high amounts of crista membrane in a more lamellar shape and high respiratory activity and ATP-generating capacity. Furthermore, the “low-performance” end of this spectrum extends into the pathological states, where swelling and disappearance of cristae are clearly visible. (See also Discussion, lines 537-548). The other measure we use besides lamellarity is crista-membrane density (surface area of crista membrane/mitochondrial volume), which is also a widely used measure for mitochondrial inner membrane architecture (Else et al., 2004; Sood et al., 2014; Strohm and Daniels, 2003). We are confident, that these objective measures are well suited to describe complex 3D morphology of mitochondrial crista structure. This is also confirmed by very detailed modeling studies, defining exactly these two measures as basic determinants of mitochondrial function (Song et al. 2013.).

Since the paragraph starting from lane 665 (whilst tighter cristae...) - 670 (more effective to produce ATP) is not relevant for the conclusions and is too speculative, I suggest that it should be omitted.

We would like to thank the Reviewer for pointing out that the statements above have not been correctly referenced in the paper. We have now inserted the exact reference for each sentence and also changed the phrase “tighter cristae” to “more lamellar cristae” as suggested. The first part of the paragraph in question is based on the work of Cogliati et al., 2013., who show and conclude that: “Taken together, these results indicate that RCS (respiratory chain supercomplex) assembly is facilitated by increased OPA1 levels and tighter cristae.” Our next sentence is based on Song et al., 2013., who show that more lamellar cristae directly lead to an increased proton motive force. Davies et al., 2011., clearly demonstrate, that ATP-synthase dimer-rows are localized to crista rims. Lamellar crista morphology contains highly curved rims with a higher possibility (lines 554-560).

o Second paragraph:

The heading and discussion has to be changed. “Ultrastructural remodelling” suggests that “low performance” synaptic boutons can be converted in “high performance” synaptic boutons and vice versa. There is no evidence and no reason for this suggestion.

The statement “the local demand for mitochondrial performance is communicated to these organelles, prompting their activity-dependent ultrastructural plasticity” does not reflect the situation. There are different types of cells with different mitochondria with different ATP production and possibly other functions. There is no “remodeling” or “ultrastructural plasticity”. The differences of the observed ultrastructures are probably the result of unknown signaling processes that sense the need for more or less respiratory complexes and communicate this to nuclear and mitochondrial transcription. Lamellar cristae provide much more space than tubular cristae and are therefore formed predominantly in these cells.

We thank the Reviewer for this clarification. In the second set of tomographic experiments we collected the samples from a well-defined glutamatergic bouton population, where the vast majority of boutons come from a single cell type, which is supported by numerous studies. The strong correlation between the studied parameters and ultrastructural determinants of synaptic strength verified the cell-type independent nature of the coupling between presynaptic mitochondrial ultrastructure and synaptic strength. As it is now discussed in the paper, there are two possible biological scenarios that could explain our observations, namely redistribution or plasticity of mitochondria. We have added the following lines to the discussion, to clarify this issue:

“Neuronal mitostasis - the maintenance of an appropriately distributed pool of healthy mitochondria - is fundamental for proper neuronal function (reviewed by Misgeld and Schwarz, 2017). Our observations verified the presence of a cell-type independent coupling between presynaptic mitochondrial ultrastructure and synaptic strength. This coupling - responsible for local demand matching - could be achieved either by a coordinated long-range retrograde/anterograde axonal trafficking of mitochondria, or by local plasticity mechanisms that lead to adaptation at the level of single organelles.

During synaptic plasticity, the energetic demand of a potentiated synapse will increase. In case of the first mechanism - organelle trafficking - the mitochondrion from the potentiated bouton would need to be withdrawn, transported away - possibly back to the soma - and another, stronger mitochondrion would be sent out to replace it. The coordination of this process, together with the evident gap in energy supply during the change is more than problematic. On the other hand, the large number of papers dealing with mitochondrial motility and redistribution performed mainly in vitro experiments, however, recent in vivo two-photon imaging studies suggest that mitochondrial motility is way much smaller, and also quite independent from neuronal activity (Lewis et al., 2016; Smit-Rigter et al., 2016). This suggests that this mechanism alone is not sufficient to account for local demand matching. The other possible mechanism is locally regulated ultrastructural plasticity. In fact, in non-neuronal tissues, an overwhelming body of evidence confirms that dynamic ultrastructural remodeling of mitochondria takes place as a response to altered energetic demand (Cogliati et al., 2016).”

Dynamic rearrangement of cristae can undergo on the timescale of minutes. We also cite recent works, confirming several molecular pathways, through which the change in metabolic demand can induce the ultrastructural plasticity of mitochondria (i.e. Patten et al.: “OPA1-dependent cristae modulation is essential for cellular adaptation to metabolic demand”). It is also well established, that local protein synthesis is required for mitochondrial function, further strengthening the idea of local regulation. We think that our reasoning and the amended discussion fits well into the current view of mitochondrial dynamics and neuroplasticity.

o Patten et al. did clearly show that the steady state level of Opa1 did not vary upon starvation, but its oligomeric state. This has to be changed in the discussion!

The discussion has been corrected accordingly.

Minor Points:

- The authors use the terms “ultrastructure”, “sub-organelle ultrastructure” and “nano-scale architecture” that have an identical meaning. This is confusing. The usual term in the literature is “ultrastructure”.

Thank you for pointing this out. We have corrected the manuscript, and use the term “ultrastructure” consequently.

- The abbreviations of buffers and their compositions should be given.

We have included the abbreviations and the exact composition of the buffers in the manuscript.

- Error: Line 553 Fig. 3-2. B should be Fig. 3-1. B.

Thank you for pointing this out, we have corrected the reference.

- It would be appropriate to show directly that the mitochondrial reconstructions provided in Fig 3 are derived from boutons with a non-perforated active zone or from boutons with a multiple-perforated active zone.

We have amended Fig3 accordingly: we labeled “LP” and “HP” boutons on the panels, and also labeled that the “HP” bouton possesses multiple active zones.

- The completely different scales in Figure 3E and 4G are confusing. I recommend to keep it use the same scale in both figures.

The panels in Fig 3E and 4G both show mitochondrial volume values, however on Fig 3 we show results from a dehydrated and plastic embedded sample, obtained through serial-EM measurement, while on Figure 4 we show results from immunolabeled tissue sample and confocal microscopy. We have corrected the scale on Figure 4, and now both panels are on the same scale in the manuscript.

Response to Reviewer #2:

REVIEWER 2

This paper is among the many that have recently begun to re-address an important overarching topic - whether/how are synaptic transmission and plasticity reliant upon mitochondrial function by means of regulation of mitochondrial shape and dynamics. This had begun to be studied decades ago, but now with new and improved structural and functional assays, the field is experiencing a resurgence. In line with this, these authors use a lovely combination of transmission EM, confocal, and localization-based microscopy to study their hypothesis that presynaptic mitochondrial ultrastructure controls mitochondrial function in order to meet synaptic needs. This manuscript tests a couple predictions of this hypothesis, specifically whether mitochondrial structure is correlated with mitochondrial function (or rather, structural readouts with inferred functional implications) and synaptic function. Furthermore, the authors test whether this is synapse specific by studying three different synapses. They also test whether this is cell-type specific by morphometrically subcategorizing synapses within one category (PP-DG) into high and low performance.

The authors' overarching conclusion is that “not only the mitochondrial nano-architecture is coupled to synaptic performance in a cell-type independent manner, but also the expression of the levels of the respiratory chain protein cytochrome c”. Furthermore, the authors contextualize their findings with the literature and state “the primary way of adjusting mitochondrial performance to the actual demand at synapses could be the activity-dependent ultrastructural plasticity of these organelles...these changes in mitochondrial nano-architecture and molecular fingerprints could contribute to neuroplasticity at the level of individual synapses”. This manuscript is an incomplete study of the large stated hypothesis. However, many of the individual conclusions within the manuscript are supported by the data presented, while only a small subset are not. Nevertheless, a couple of small additions would help clarify the conclusions presented.

- In 1 H - CM density is higher in PV. But, crista width seems to be greater in CB1R. Does this have any putative functional relevance? Refer to HL Smith et al, eLife 2016.

- It would be helpful if the authors could explain why the CM density is an appropriate measure here. Cogliati et al, 2013 suggest blebbier cristae appear approaching the “apoptotic” spectrum and thus these mitos have less respiratory functional capacity.

- In addition to CM density and crista shape factor, the authors could also measure cristae width, which was recently (HL Smith et al, eLife 2016) correlated with an increase in presynaptic function following TBS-LTP. Is cristae width also greater in HP/PV compared to LP/CB1R boutons?

We would like to thank the Reviewer for these questions and suggestions. The first parameter we use is crista-membrane density (surface area of crista membrane/mitochondrial volume), which is a widely used measure to describe mitochondrial inner membrane architecture. On the other hand, a lot of papers dealing with mitochondrial crista morphology also use the measure of crista width. However, this way they are using a 2-dimensional value to give a simplified representation of a 3D feature. To overcome this problem, we introduced “crista shape factor” (surface area of crista membrane/crista lumen volume), enabling us to objectively describe the lamellarity (tightness) of mitochondrial cristae in the 3D-space using electron tomography datasets. In fact, a very elegant modeling study defines exactly these two measures as basic determinants of mitochondrial function (Song et al. 2013., referenced in our manuscript), confirming, that these objective measures are well suited to describe complex 3D morphology of mitochondrial crista structure. The latest studies in the literature and our observations also suggest that there is a continuous spectrum of mitochondrial ultrastructure, ranging from mitochondria with lower crista membrane density, less lamellar cristae and lower respiratory activity to mitochondria possessing high amounts of crista membrane in a more lamellar shape and high respiratory activity and ATP-generating capacity. Furthermore, the “low-performance” end of this spectrum extends into the pathological states, where swelling and disappearance of cristae are clearly visible. The vast majority of recent studies are thus contradicting Hackenbrock's measurements from the 1960-s. Hackenbrock - using an ex vivo mitochondrial preparation - concluded that larger crista width (and thus less lamellar cristae) are beneficial for respiration. However, those observations have not been verified under physiological conditions, and since then, a large number of independent groups - utilizing very precise methodology - showed that the opposite is the case.

In the context of crista width, more lamellar cristae would mean smaller values. According to this, we would expect that crista width would be smaller in PV/HP-boutons, than in CB1/LP-ones. To test this, we performed the 2D measurements of crista width on our datasets. We found, that mitochondria in PV boutons had a crista width of 27.69 nm (median, 25.93-29.46 interquartile range), while this value was 42.49 nm (median, 38.33-44.07 interquartile range) in CB1 boutons. This difference was significant with a p-value of 0. 00018 (Mann-Whitney U-test). We also performed these measurements on glutamatergic boutons, and found that mitochondria in HP boutons had a crista width of 36.58 nm (median, 30.09-38.7 interquartile range), while this value was 54.19 nm (median, 44.52-59.17 interquartile range) in LP boutons. This difference was also significant with a p-value of 0. 0017 (Mann-Whitney U-test). These results agree with our crista lamellarity measurements but due to the argument above, we think these data would not add novel information to the manuscript.

In their recent paper HL Smith et al. evoked LTP with theta burst stimulation (TBS) using in vitro slices, but did not check whether the mitochondria they studied are located at potentiated synapses or possibly at newly generated synapses. In this context, we can only conclude from their measurements that in an in vitro slice preparation TBS leads to an elevated number of mitochondria with wider cristae. Our findings - by correlating mitochondrial ultrastructure and synaptic morphology at individual active zones - support the current view, i.e. the higher the crista membrane density is, and the lamellar the cristae are (the smaller the crista width is), the higher the mitochondrial performance is.

- Comparing even a few HP and LP boutons from the same axon (similar to HL Smith et al., eLife 2016, and others) would strengthen the conclusion. Is there a way to do this in the existing data?

We agree that it would be valuable to compare different mitochondria within a single axon and link their anatomical features with synaptic performance. However, in these measurements, we performed dual-axis electron tomography on EM grids that had been subjected to serial-section TEM analysis previously. Dual-axis tomography is not only a very labour-intensive process, it is also very easy to damage a grid during repeated image acquisition and other technical issues (e.g. the necessity to manually rotate the grid before the acquisition of the second axis tilt-series) also limit the number of acquisitions using the same biological sample. Thus, to find even a few LP/HP boutons from the same axon would require such an amount of work that we felt this would be beyond the scope of this paper and would substantially delay publication of these extensive data sets. Instead, we collected the samples from a well-defined glutamatergic bouton population, and the strong correlation between the studied parameters and ultrastructural determinants of synaptic strength verified the cell-type independent nature of the ultrastructural coupling between presynaptic mitochondria and synaptic strength.

- The sentence “These results suggest a strong correlation between the ultrastructure of presynaptic mitochondria and the performance of synapses” is perhaps overstated. All the authors can conclude thus far is that mitochondria within two different populations of basket cell boutons are ultrastructurally different. Perhaps “presumed performance” or some such minor qualifier?

We have corrected the text accordingly.

- For Fig 2, authors should clarify that they mean that the BORDERS of the Tom20 and CytC are overlapping, not that the labeled protein points overlap. And from a technical standpoint, it is not clear (perhaps I missed a detail in the methods) whether the points shown for the staining are those that passed some set of criteria to help define the mitochondria or if that is all the localizations from the staining. Are the points shown after processing or “raw data”?

Thank you for drawing our attention to this important issue. We have corrected the text accordingly, and clarified that we found a near-complete overlap between the Tom20 and CytC-labeled areas, and not among STORM localization points of the two channels. We have also amended the methods section accordingly, and inserted: “Localisation points exceeding a given photon count were counted as specific SLPs.”

- The authors state that “the average HP bouton utilizes 4.67 times more mitochondrial crista membrane surface than the average LP bouton”. Without a functional assay, the use of the term “utilizes” seems inappropriate.

We replaced the term “utilizes” with “possesses”.

- Was the image quality high enough in the human sample to analyze CM density or crista shape? Those values would be useful to include.

Please see also our response to a similar query of Reviewer 1 above.

Figures-

- It would be helpful to have text in the panel stating which bouton type we're observing. This comment is applicable to the first panels of each of the 4 figures. Similarly, I'm not a fan of the gray background in the graphs, which decreases contrast and readability.

We thank the Reviewer for these suggestions; we have now displayed bouton types (PV vs. CB1 and LP vs. HP) in the figure panels, and also changed the grey background to a lighter tone for better visibility.

- Axis labels and units are missing in Fig. 2B.

We corrected the figure accordingly, and displayed the unit.

- Figure 1A says CitC instead of CytC.

Thank you very much, we corrected Fig 4A.

## References

[B1] Amaral DG, Scharfman HE, Lavenex P (2007) The dentate gyrus: fundamental neuroanatomical organization (dentate gyrus for dummies). Prog Brain Res 163:3–22. 17765709 10.1016/S0079-6123(07)63001-5 PMC2492885

[B2] Andreska T, Aufmkolk S, Sauer M, Blum R (2014) High abundance of BDNF within glutamatergic presynapses of cultured hippocampal neurons. Front Cell Neurosci 8:107 10.3389/fncel.2014.00107 24782711 PMC3990111

[B3] Aschrafi A, Schwechter AD, Mameza MG, Natera-Naranjo O, Gioio AE, Kaplan BB (2008) MicroRNA-338 regulates local cytochrome c oxidase IV mRNA levels and oxidative phosphorylation in the axons of sympathetic neurons. J Neurosci 28:12581–12590. 10.1523/JNEUROSCI.3338-08.2008 19020050 PMC3496265

[B4] Barna L, Dudok B, Miczán V, Horváth A, László ZI, Katona I (2016) Correlated confocal and super-resolution imaging by VividSTORM. Nat Protoc 11:163–183. 10.1038/nprot.2016.002 26716705

[B5] Barnhart EL (2016) Mechanics of mitochondrial motility in neurons. Curr Opin Cell Biol 38:90–99. 26986984 10.1016/j.ceb.2016.02.022

[B6] Billups B, Forsythe ID (2002) Presynaptic mitochondrial calcium sequestration influences transmission at mammalian central synapses. J Neurosci 22:5840–5847. 12122046 10.1523/JNEUROSCI.22-14-05840.2002 PMC6757942

[B7] Bourne JN, Harris KM (2012) Nanoscale analysis of structural synaptic plasticity. Curr Opin Neurobiol 22:372–382. 10.1016/j.conb.2011.10.019 22088391 PMC3292623

[B8] Buchs PA, Muller D (1996) Induction of long-term potentiation is associated with major ultrastructural changes of activated synapses. Proc Natl Acad Sci USA 93:8040–8045. 10.1073/pnas.93.15.8040 8755599 PMC38871

[B9] Chandel NS (2014) Mitochondria as signaling organelles. BMC Biol 12 10.1186/1741-7007-12-34 PMC4035690 24884669

[B10] Cheetham CEJ, Barnes SJ, Albieri G, Knott GW, Finnerty GT (2014) Pansynaptic enlargement at adult cortical connections strengthened by experience. Cereb Cortex 24:521–531. 10.1093/cercor/bhs334 23118196 PMC3888373

[B11] Choi KJ, Kim MJ, Je AR, Jun S, Lee C, Lee E, Jo M, Huh YH, Kweon HS (2014) Three-dimensional analysis of abnormal ultrastructural alteration in mitochondria of hippocampus of APP/PSEN1 transgenic mouse. J Biosci 39:97–105. 24499794 10.1007/s12038-013-9406-8

[B12] Civiletto G, Varanita T, Cerutti R, Gorletta T, Barbaro S, Marchet S, Lamperti C, Viscomi C, Scorrano L, Zeviani M (2015) Opa1 overexpression ameliorates the phenotype of two mitochondrial disease mouse models. Cell Metab 21:845–854. 26039449 10.1016/j.cmet.2015.04.016 PMC4457891

[B13] Cogliati S, Frezza C, Soriano ME, Varanita T, Quintana-Cabrera R, Corrado M, Cipolat S, Costa V, Casarin A, Gomes LC, Perales-Clemente E, Salviati L, Fernandez-Silva P, Enriquez JA, Scorrano L (2013) Mitochondrial cristae shape determines respiratory chain supercomplexes assembly and respiratory efficiency. Cell 155:160–171. 24055366 10.1016/j.cell.2013.08.032 PMC3790458

[B14] Cogliati S, Enriquez JA, Scorrano L (2016) Mitochondrial cristae: where beauty meets functionality. Trends Biochem Sci 41:261–273. 26857402 10.1016/j.tibs.2016.01.001

[B15] Daum B, Walter A, Horst A, Osiewacz HD, Kühlbrandt W (2013) Age-dependent dissociation of ATP synthase dimers and loss of inner-membrane cristae in mitochondria. Proc Natl Acad Sci USA 110:15301–15306. 10.1073/pnas.1305462110 24006361 PMC3780843

[B16] Davies KM, Strauss M, Daum B, Kief JH, Osiewacz HD, Rycovska A, Zickermann V, Kühlbrandt W (2011) Macromolecular organization of ATP synthase and complex I in whole mitochondria. Proc Natl Acad Sci USA 108:14121–14126. 10.1073/pnas.1103621108 21836051 PMC3161574

[B17] Desmond NL, Levy WB (1988) Synaptic interface surface area increases with long-term potentiation in the hippocampal dentate gyrus. Brain Res 453:308–314. 3401768 10.1016/0006-8993(88)90171-0

[B18] Dikov D, Bereiter-Hahn J (2013) Inner membrane dynamics in mitochondria. J Struct Biol 183:455–466. 23792165 10.1016/j.jsb.2013.06.003

[B19] Else PL, Turner N, Hulbert AJ (2004) The evolution of endothermy: role for membranes and molecular activity. Physiol Biochem Zool 77:950–958. 10.1086/422767 15674769

[B20] Gale JR, Aschrafi A, Gioio AE, Kaplan BB (2017) Nuclear-encoded mitochondrial mRNAs: a powerful force in axonal growth and development. Neuroscientist 107385841771422. 10.1177/1073858417714225 28614981

[B21] Gazit N, Vertkin I, Shapira I, Helm M, Slomowitz E, Sheiba M, Mor Y, Rizzoli S, Slutsky I (2016) IGF-1 receptor differentially regulates spontaneous and evoked transmission via mitochondria at hippocampal synapses. Neuron 89:583–597. 26804996 10.1016/j.neuron.2015.12.034 PMC4742535

[B22] Geinisman Y (2000) Structural synaptic modifications associated with hippocampal LTP and behavioral learning. Cereb Cortex 10:952–962. 10.1093/cercor/10.10.952 11007546

[B23] Gomes LC, Di Benedetto G, Scorrano L (2011) During autophagy mitochondria elongate, are spared from degradation and sustain cell viability. Nat Cell Biol 13:589–598. 10.1038/ncb2220 21478857 PMC3088644

[B24] Gulyás AI, Buzsáki G, Freund TF, Hirase H (2006) Populations of hippocampal inhibitory neurons express different levels of cytochrome c. Eur J Neurosci 23:2581–2594. 16817861 10.1111/j.1460-9568.2006.04814.x

[B25] Gunter TE, Buntinas L, Sparagna G, Eliseev R, Gunter K (2000) Mitochondrial calcium transport: mechanisms and functions. Cell Calcium 28:285–296. 10.1054/ceca.2000.0168 11115368

[B26] Hackenbrock CR (1966) Ultrastructural bases for metabolically linked mechanical activity in mitochondria. I. Reversible ultrastructural changes with change in metabolic steady state in isolated liver mitochondria. J Cell Biol 30:269–297. 10.1083/jcb.30.2.269 5968972 PMC2107001

[B27] Hahn A, Parey K, Bublitz M, Mills DJ, Zickermann V, Vonck J, Kühlbrandt W, Meier T (2016) Structure of a complete ATP synthase dimer reveals the molecular basis of inner mitochondrial membrane morphology. Mol Cell 63:445–456. 27373333 10.1016/j.molcel.2016.05.037 PMC4980432

[B28] Hall CN, Klein-Flugge MC, Howarth C, Attwell D (2012) Oxidative phosphorylation, not glycolysis, powers presynaptic and postsynaptic mechanisms underlying brain information processing. J Neurosci 32:8940–8951. 10.1523/JNEUROSCI.0026-12.2012 22745494 PMC3390246

[B29] Hambrecht R, Fiehn E, Yu J, Niebauer J, Weigl C, Hilbrich L, Adams V, Riede U, Schuler G (1997) Effects of endurance training on mitochondrial ultrastructure and fiber type distribution in skeletal muscle of patients with stable chronic heart failure. J Am Coll Cardiol 29:1067–1073. 10.1016/S0735-1097(97)00015-6 9120161

[B30] Harner M, Körner C, Walther D, Mokranjac D, Kaesmacher J, Welsch U, Griffith J, Mann M, Reggiori F, Neupert W (2011) The mitochondrial contact site complex, a determinant of mitochondrial architecture. EMBO J 30:4356–4370. 10.1038/emboj.2011.379 22009199 PMC3230385

[B31] Harris JJ, Jolivet R, Attwell D (2012) Synaptic energy use and supply. Neuron 75:762–777. 22958818 10.1016/j.neuron.2012.08.019

[B32] Hillefors M, Gioio AE, Mameza MG, Kaplan BB (2007) Axon viability and mitochondrial function are dependent on local protein synthesis in sympathetic neurons. Cell Mol Neurobiol 27:701–716. 17619140 10.1007/s10571-007-9148-y PMC11517218

[B33] Holderith N, Lorincz A, Katona G, Kulik A, Watanabe M, Nusser Z (2012) Release probability of hippocampal glutamatergic terminals scales with the size of the active zone. Nat Neurosci 15:988–997. 22683683 10.1038/nn.3137 PMC3386897

[B34] Holtmaat A, Svoboda K (2009) Experience-dependent structural synaptic plasticity in the mammalian brain. Nat Rev Neurosci 10:759–759. 10.1038/nrn2721 19693029

[B35] Huang B, Jones SA, Brandenburg B, Zhuang X (2008) Whole-cell 3D STORM reveals interactions between cellular structures with nanometer-scale resolution. Nat Methods 5:1047–1052. 10.1038/nmeth.1274 19029906 PMC2596623

[B36] Ivannikov MV, Sugimori M, Llinás RR (2013) Synaptic vesicle exocytosis in hippocampal synaptosomes correlates directly with total mitochondrial volume. J Mol Neurosci 49:223–230. 22772899 10.1007/s12031-012-9848-8 PMC3488359

[B37] Jimenez L, Laporte D, Duvezin-Caubet S, Courtout F, Sagot I (2014) Mitochondrial ATP synthases cluster as discrete domains that reorganize with the cellular demand for oxidative phosphorylation. J Cell Sci 127:719–726. 10.1242/jcs.137141 24338369

[B38] Kann O, Papageorgiou IE, Draguhn A (2014) Highly energized inhibitory interneurons are a central element for information processing in cortical networks. J Cereb Blood Flow Metab 34:1270–1282. 10.1038/jcbfm.2014.104 24896567 PMC4126088

[B39] Klausberger T, Marton LF, O’Neill J, Huck JHJ, Dalezios Y, Fuentealba P, Suen WY, Papp E, Kaneko T, Watanabe M, Csicsvari J, Somogyi P (2005) Complementary roles of cholecystokinin- and parvalbumin-expressing GABAergic neurons in hippocampal network oscillations. J Neurosci 25:9782–9793. 16237182 10.1523/JNEUROSCI.3269-05.2005 PMC6725722

[B40] Korogod N, Petersen CCH, Knott GW (2015) Ultrastructural analysis of adult mouse neocortex comparing aldehyde perfusion with cryo fixation. Elife 4. 10.7554/eLife.05793 PMC4530226 26259873

[B41] Kremer JR, Mastronarde DN, Mcintosh JR (1996) Computer visualization of three-dimensional image data using IMOD. J Struct Biol 116:71–76. 10.1006/jsbi.1996.0013 8742726

[B42] Lapray D, Lasztoczi B, Lagler M, James Viney T, Katona L, Valenti O, Hartwich K, Borhegyi Z, Somogyi P, Klausberger T (2012) Behavior-dependent specialization of identified hippocampal interneurons. Nat Neurosci 15 10.1038/nn.3176 PMC3433735 22864613

[B43] Lewis TL, Turi GF, Kwon SK, Losonczy A, Polleux F (2016) Progressive decrease of mitochondrial motility during maturation of cortical axons *in vitro* and *in vivo*. Curr Biol 26:2602–2608. 10.1016/j.cub.2016.07.064 27641765 PMC5235338

[B44] Li Z, Okamoto KI, Hayashi Y, Sheng M (2004) The importance of dendritic mitochondria in the morphogenesis and plasticity of spines and synapses. Cell 119:873–887. 15607982 10.1016/j.cell.2004.11.003

[B45] Macchi M, El Fissi N, Tufi R, Bentobji M, Liévens JC, Martins LM, Royet J, Rival T (2013) The *Drosophila* inner-membrane protein PMI controls crista biogenesis and mitochondrial diameter. J Cell Sci 126:814–824. 10.1242/jcs.115675 23264743

[B46] Mannella CA, Lederer WJ, Jafri MS (2013) The connection between inner membrane topology and mitochondrial function. J Mol Cell Cardiol 62:51–57. 10.1016/j.yjmcc.2013.05.001 23672826 PMC4219563

[B47] Melone M, Burette A, Weinberg RJ (2005) Light microscopic identification and immunocytochemical characterization of glutamatergic synapses in brain sections. J Comp Neurol 492:495–509. 16228991 10.1002/cne.20743

[B48] Misgeld T, Schwarz TL (2017) Mitostasis in neurons: maintaining mitochondria in an extended cellular architecture. Neuron 96:651–666. 10.1016/j.neuron.2017.09.055 29096078 PMC5687842

[B49] Muller D, Nikonenko I, Jourdain P, Alberi S (2002) LTP, memory and structural plasticity. Curr Mol Med 2:605–611. 12420800 10.2174/1566524023362041

[B50] Neupert W (2012) SnapShot: mitochondrial architecture. Cell 149:722–722.e1. 10.1016/j.cell.2012.04.010 22541440

[B51] Nicastro D, Frangakis AS, Typke D, Baumeister W (2000) Cryo-electron tomography of neurospora mitochondria. J Struct Biol 129:48–56. 10.1006/jsbi.1999.4204 10675296

[B52] Nielsen J, Gejl KD, Hey-mogensen M, Holmberg H-C, Suetta C, Krustrup P, Elemans CPH, Ørtenblad N (2017) Plasticity in mitochondrial cristae density allows metabolic capacity modulation in human skeletal muscle. J Physiol 595:2839–2847. 27696420 10.1113/JP273040 PMC5407961

[B53] Patten DA, Wong J, Khacho M, Soubannier V, Mailloux RJ, Pilon-larose K, Maclaurin JG, Park DS, Mcbride HM, Trinkle-Mulcahy L, Harper ME, Germain M, Slack RS (2014) OPA 1-dependent cristae modulation is essential for cellular adaptation to metabolic demand. EMBO J 33:2676–2691. 10.15252/embj.201488349 25298396 PMC4282575

[B54] Perkins G, Hsiao Y, Yin S, Tjong J, Tran MT, Lau J, Xue J, Liu S, Ellisman MH, Zhou D (2012) Ultrastructural modifications in the mitochondria of hypoxia-adapted *Drosophila* melanogaster. PLoS One 7:e45344. 23028948 10.1371/journal.pone.0045344 PMC3446896

[B55] Perkins GA, Song JY, Tarsa L, Deerinck TJ, Ellisman MH, Frey TG (1998) Electron tomography of mitochondria from brown adipocytes reveals crista junctions. J Bioenerg Biomembr 30:431–442. 9932646 10.1023/a:1020586012561

[B56] Perkins GA, Ellisman MH, Fox DA (2003) Three-dimensional analysis of mouse rod and cone mitochondrial cristae architecture: bioenergetic and functional implications. Mol Vis 9:60–73. 12632036

[B57] Popov VI, Davies HA, Rogachevsky VV, Patrushev IV, Errington ML, Gabbott PLA, Bliss TVP, Stewart MG (2004) Remodelling of synaptic morphology but unchanged synaptic density during late phase long-term potentiation (LTP): a serial section electron micrograph study in the dentate gyrus in the anaesthetised rat. Neuroscience 128:251–262. 15350638 10.1016/j.neuroscience.2004.06.029

[B58] Rangaraju V, Calloway N, Ryan TA (2014) Activity-driven local ATP synthesis is required for synaptic function. Cell 156:825–835. 10.1016/j.cell.2013.12.042 24529383 PMC3955179

[B59] Sheng ZH (2014) Mitochondrial trafficking and anchoring in neurons: new insight and implications. J Cell Biol 204:1087–1098. 10.1083/jcb.201312123 24687278 PMC3971748

[B60] Smit-Rigter L, Rajendran R, Silva CAP, Spierenburg L, Groeneweg F, Ruimschotel EM, van Versendaal D, van der Togt C, Eysel UT, Heimel JA, Lohmann C, Levelt CN (2016) Mitochondrial dynamics in visual cortex are limited *in vivo* and not affected by axonal structural plasticity. Curr Biol 26:2609–2616. 10.1016/j.cub.2016.07.033 27641766

[B61] Smith HL, Bourne JN, Cao G, Chirillo MA, Ostroff LE, Watson DJ, Harris KM (2016) Mitochondrial support of persistent presynaptic vesicle mobilization with age-dependent synaptic growth after LTP. Elife 5:e15275. 10.7554/eLife.15275 27991850 PMC5235352

[B62] Song DH, Park J, Maurer LL, Lu W, Philbert MA, Sastry AM (2013) Biophysical significance of the inner mitochondrial membrane structure on the electrochemical potential of mitochondria. Phys Rev E Stat Nonlin Soft Matter Phys 88:062723. 24483502 10.1103/PhysRevE.88.062723 PMC4315510

[B63] Sood A, Jeyaraju DV, Prudent J, Caron A, Lemieux P, McBride HM, Laplante M, Tóth K, Pellegrini L (2014) A Mitofusin-2-dependent inactivating cleavage of Opa1 links changes in mitochondria cristae and ER contacts in the postprandial liver. Proc Natl Acad Sci USA 111:16017–16022. 10.1073/pnas.1408061111 25352671 PMC4234614

[B64] Strohm E, Daniels W (2003) Ultrastructure meets reproductive success: performance of a sphecid wasp is correlated with the fine structure of the flight-muscle mitochondria. Proc Biol Sci 270:749–754. 12713750 10.1098/rspb.2002.2282 PMC1691295

[B65] Sun T, Qiao H, Pan PY, Chen Y, Sheng ZH (2013) Motile axonal mitochondria contribute to the variability of presynaptic strength. Cell Rep 4:413–419. 23891000 10.1016/j.celrep.2013.06.040 PMC3757511

[B66] Takács VT, Sźónyi A, Freund TF, Nyiri G, Gulyás AI (2015) Quantitative ultrastructural analysis of basket and axo-axonic cell terminals in the mouse hippocampus. Brain Struct. Funct 220:919–940. 24407853 10.1007/s00429-013-0692-6

[B67] Toussaint C, Kugler P (1989) Anatomy and embryology morphometric analysis of mitochondria and boutons in the dentate gyrus molecular layer of aged rats. Anat Embryol 179:411–414. 10.1007/BF00305068 2735535

[B68] Verstreken P, Ly CV, Venken KJT, Koh TW, Zhou Y, Bellen HJ (2005) Synaptic mitochondria are critical for mobilization of reserve pool vesicles at *Drosophila* neuromuscular junctions. Neuron 47:365–378. 16055061 10.1016/j.neuron.2005.06.018

[B69] Völgyi K, Gulyássy P, Háden K, Kis V, Badics K, Kékesi KA, Simor A, Györffy B, Tóth EA, Lubec G, Juhász G, Dobolyi A (2015) Synaptic mitochondria: a brain mitochondria cluster with a specific proteome. J Proteomics 120:142–157. 25782751 10.1016/j.jprot.2015.03.005

[B70] Wilson DF, Harrison DK, Vinogradov A (2014) Mitochondrial cytochrome c oxidase and control of energy metabolism: measurements in suspensions of isolated mitochondria. J Appl Physiol 117:1424–1430. 10.1152/japplphysiol.00736.2014 25324517

[B71] Zimmer A, Zimmer AM, Hohmann AG, Herkenham M, Bonner TI (1999) Increased mortality, hypoactivity, and hypoalgesia in cannabinoid CB1 receptor knockout mice. Proc Natl Acad Sci USA 96:5780–5785. 10318961 10.1073/pnas.96.10.5780 PMC21937

