# Supplementary figures and images for: Mitochondrial Ultrastructure Is Coupled to Synaptic Performance at Axonal Release Sites
Source: eNeuro. 2018 Jan 29;5(1):ENEURO.0390-17.2018. doi: 10.1523/ENEURO.0390-17.2018 (PMC5788698; doi:10.1523/ENEURO.0390-17.2018)

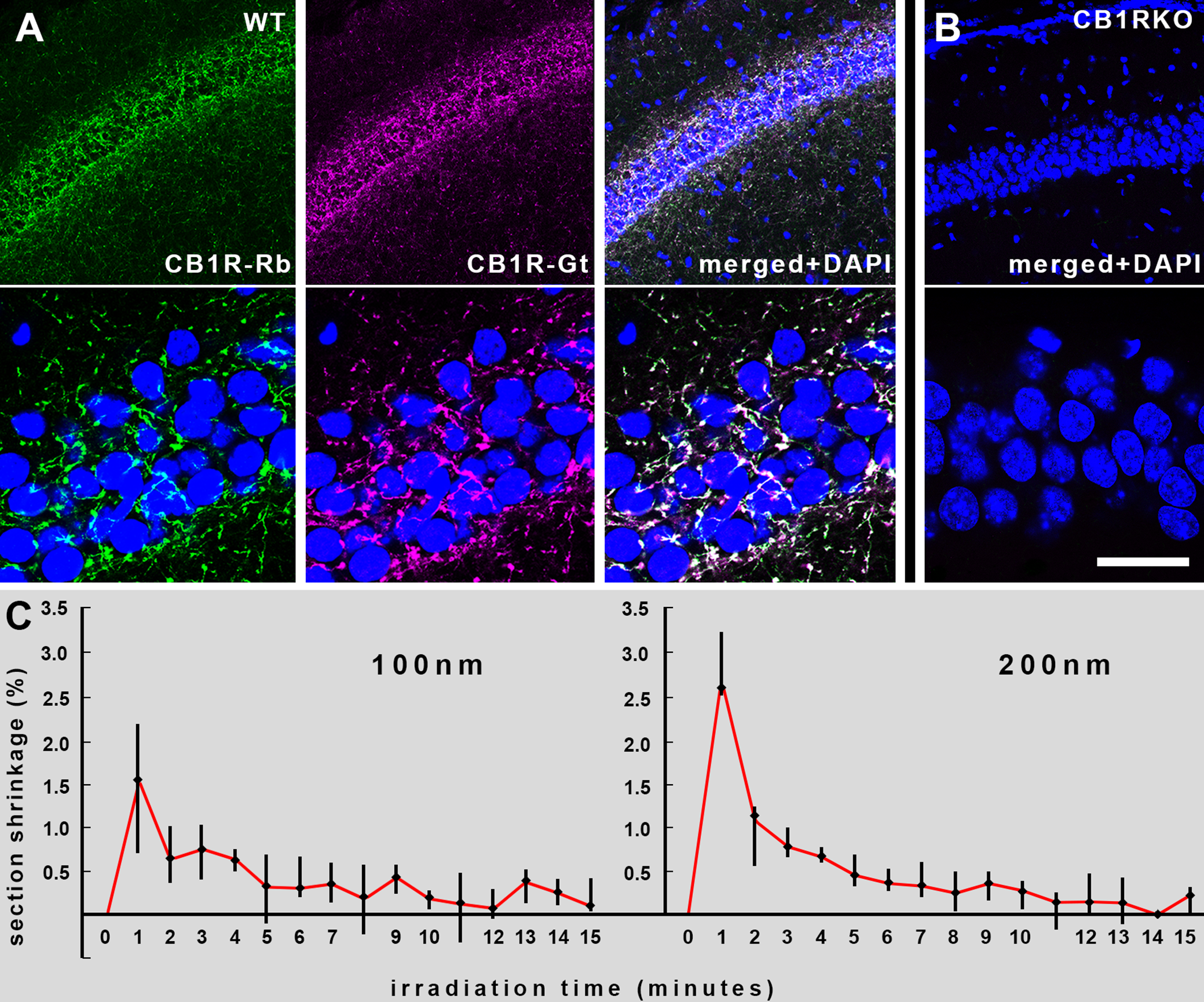

Supplement: Extended Data Figure 1-1 — Staining with two anti-CB1R antibodies is overlapping in WT, but totally absent from CB1RKO mice; rate of tissue shrinkage due to electron beam irradiation. A, Triple-color confocal images show CB1R staining in the hippocampal CA1 region of a WT mouse with a rabbit (green) and a goat (magenta) antibody. Cell nuclei are stained with DAPI (blue). B, Staining with both antibodies are completely absent in CB1R-KO mice. Scale bar: 100 µm (upper panels) and 30 µm (lower panels). C, Rates of tissue shrinkage on 100-nm-thick (left) and 200-nm-thick (right) sections under 15 min of electron beam irradiation. Black bars represent interquartile ranges, black dots mark median values. Download Figure 1-1, TIF file. [file sup_enu-eN-NWR-0390-17-s03.tif]

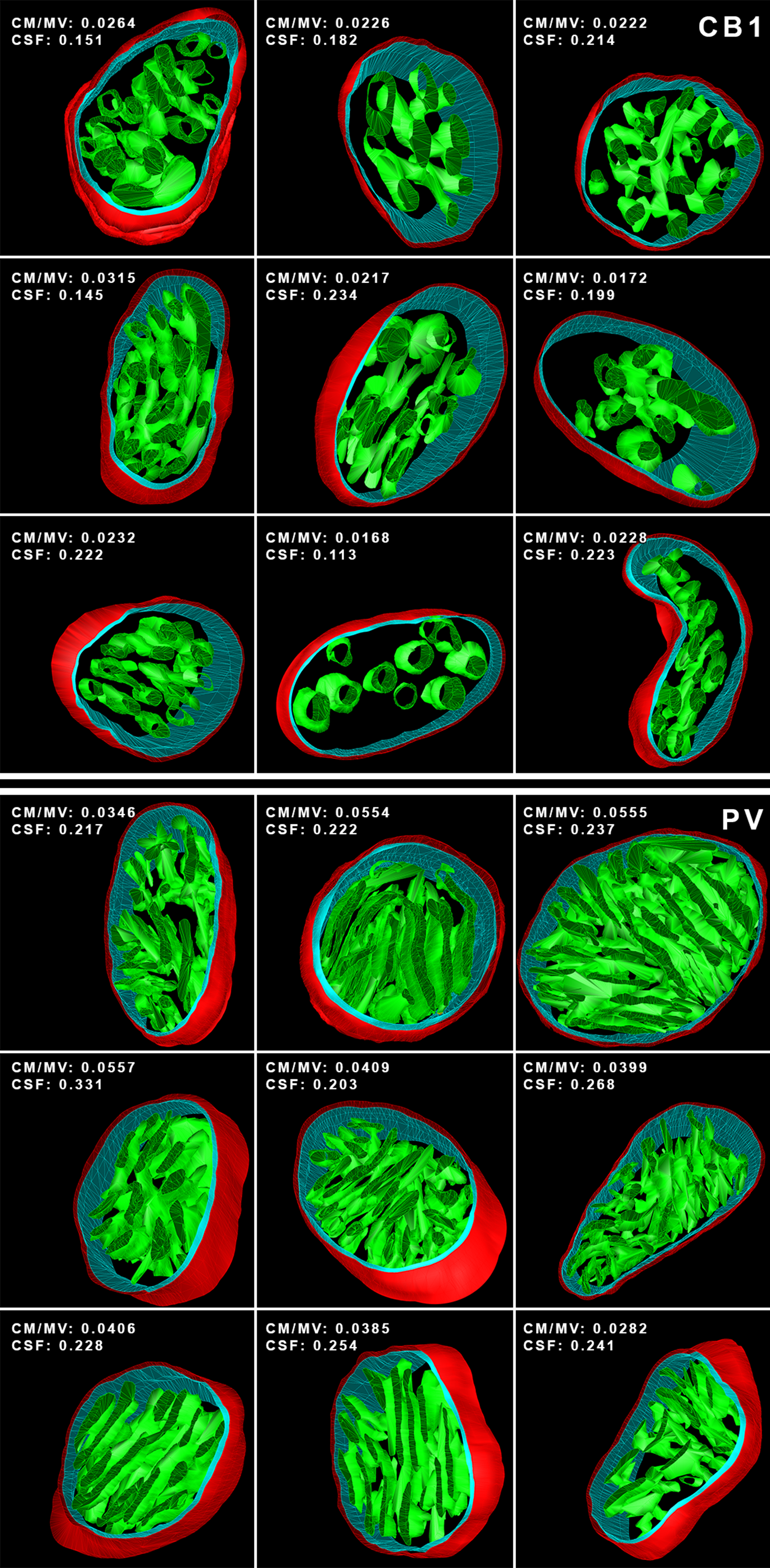

Supplement: Extended Data Figure 1-2 — 3D models of the reconstructed mitochondrial volumes from the hippocampal CA1 region. Top three rows show presynaptic mitochondria from regular spiking basket cell boutons, while the bottom three rows from fast-spiking basket-cell boutons. Mitochondrial outer membrane is red, inner boundary membrane is turquoise, CM is green. CM/MV, CM area/mitochondrial volume; CSF, crista shape factor. The models are not displayed on the same magnification. Download Figure 1-2, TIF file. [file sup_enu-eN-NWR-0390-17-s04.tif]

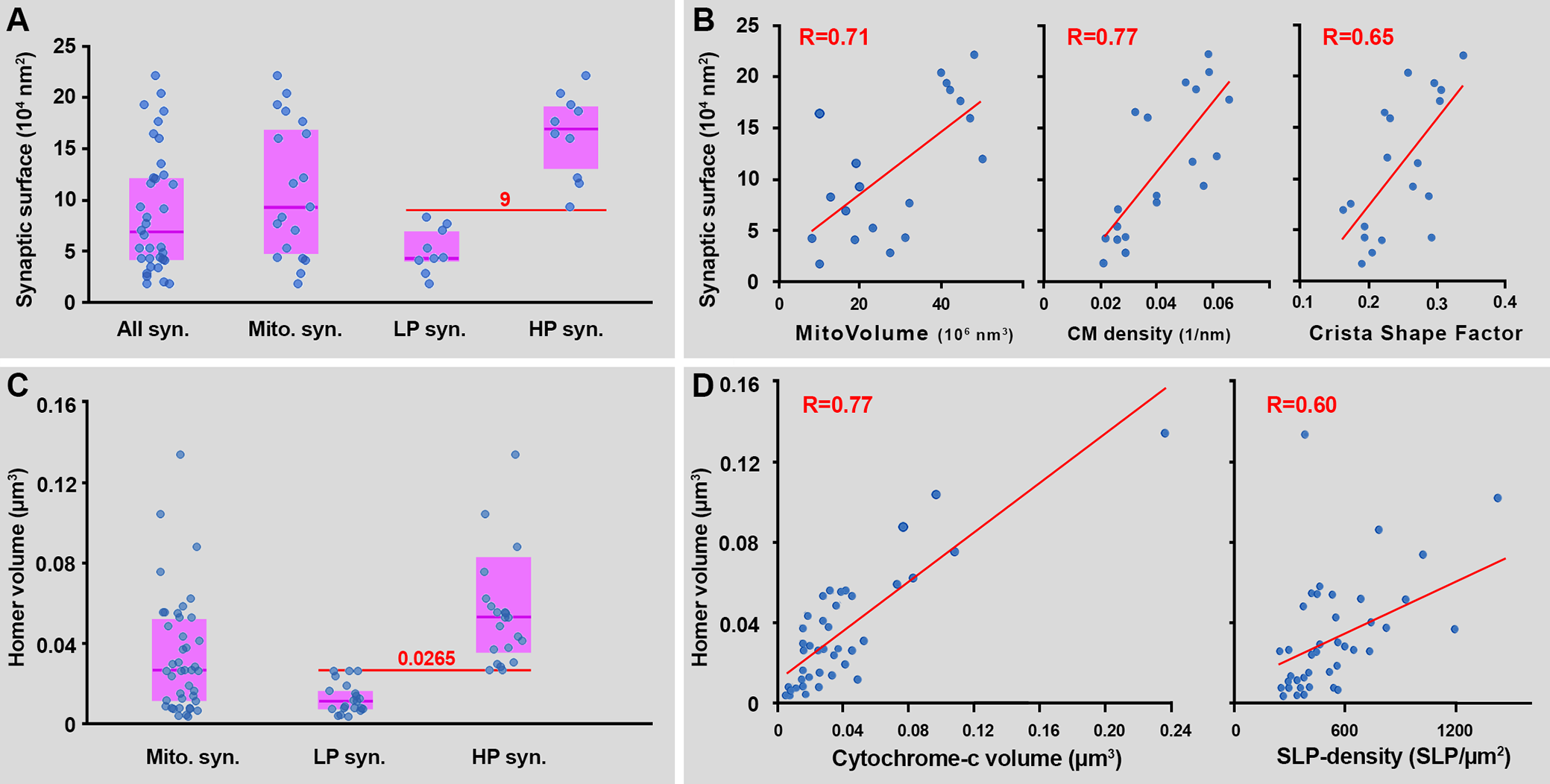

Supplement: Extended Data Figure 3-1 — Distribution of active zone sizes of the glutamatergic boutons in the tomographic and STORM measurements, and correlations of performance-determining mitochondrial features with synapse size. A, Active zone area distribution of the glutamatergic boutons examined with serial EM and electron tomography. B, The volume, CM density, and crista shape factor of presynaptic mitochondria are all strongly correlated to synapse size (R = 0.71, p = 0.0005; R = 0.77, p = 0.0001; and R = 0.65, p = 0.0026, respectively). Blue dots represent values from individual mitochondria, regression lines are red. C, Active zone area distribution of the glutamatergic boutons examined with CLSM and STORM super-resolution microscopy. All syn., all examined synaptic boutons; Mito. syn., synaptic boutons with mitochondria; LP syn., LP synaptic boutons; HP syn., HP synaptic boutons. Blue dots represent values from individual boutons, magenta rectangles represent interquartile ranges, deep-magenta lines mark median values. Red lines show values dividing HP and LP synaptic boutons. D The CytC-labeled volume and SLP density are both strongly correlated to synapse size (R = 0.77, p < 0.00001; R = 0.60, p = 0.00002, respectively). Blue dots represent values from individual mitochondria, regression lines are red. Download Figure 3-1, TIF file. [file sup_enu-eN-NWR-0390-17-s05.tif]

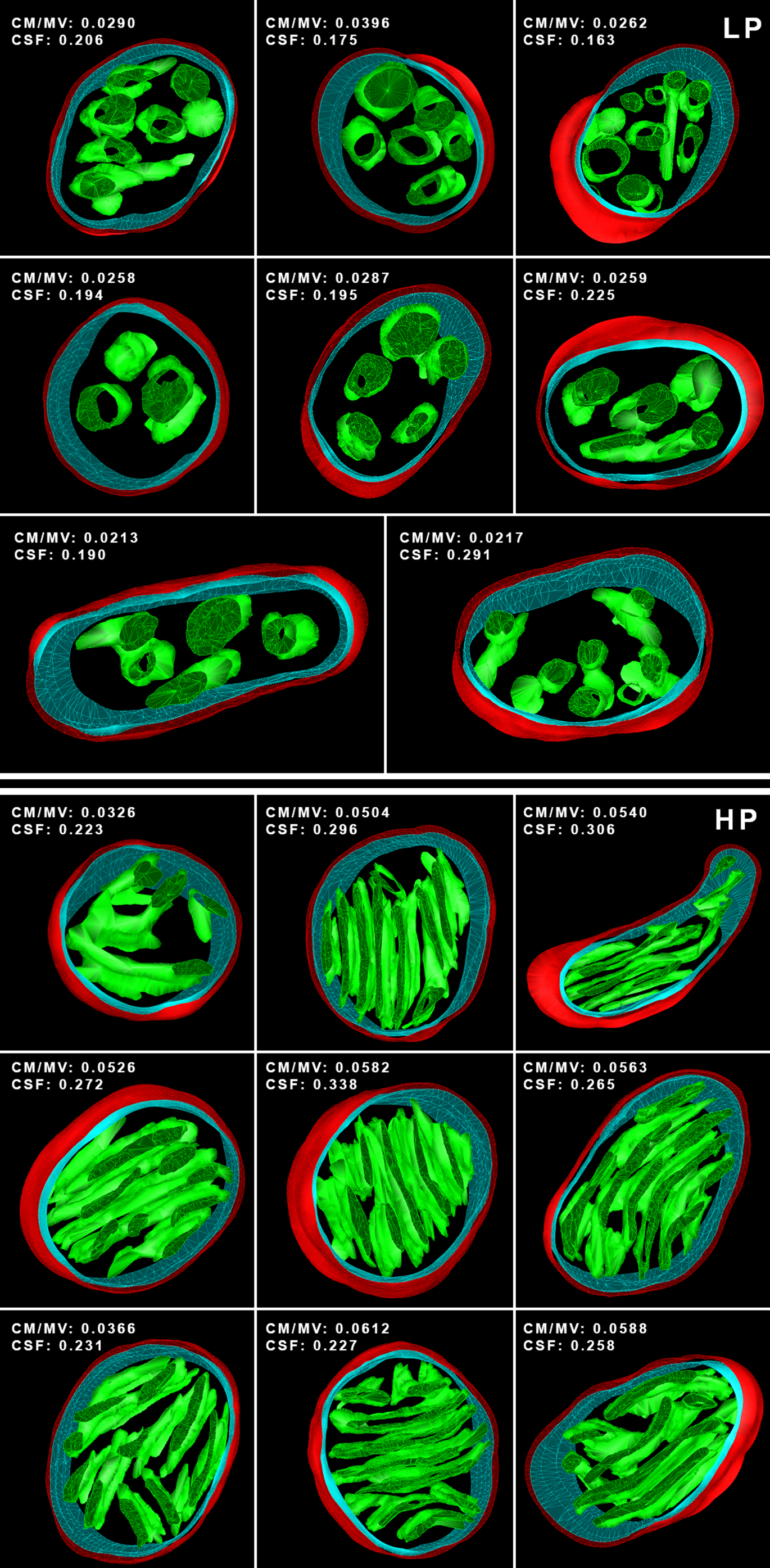

Supplement: Extended Data Figure 3-2 — 3D models of the reconstructed mitochondrial volumes from the dentate gyrus. Top three rows show presynaptic mitochondria from LP glutamatergic boutons, while the bottom three rows from HP ones. Mitochondrial outer membrane is red, inner boundary membrane is turquoise, CM is green. CM/MV, CM area/mitochondrial volume; CSF, crista shape factor. The models are not displayed on the same magnification. Download Figure 3-2, TIF file. [file sup_enu-eN-NWR-0390-17-s06.tif]
